# Structural analysis of an *Asterias rubens* peptide indicates the presence of a disulfide‐directed β‐hairpin fold

**DOI:** 10.1002/2211-5463.13931

**Published:** 2024-11-19

**Authors:** Rozita Takjoo, David T. Wilson, Justine Le Quilliec, Casey A. Schmidt, Guangzu Zhao, Michael J. Liddell, Naeem Y. Shaikh, Kartik Sunagar, Alex Loukas, Michael J. Smout, Norelle L. Daly

**Affiliations:** ^1^ Australian Institute of Tropical Health and Medicine James Cook University Cairns Australia; ^2^ Univ Brest, École Supérieure d'Ingénieurs en Agroalimentaire de Bretagne atlantique Plouzané France; ^3^ College of Science and Engineering James Cook University Cairns Australia; ^4^ Evolutionary Venomics Lab, Centre for Ecological Sciences Indian Institute of Science Bangalore India

**Keywords:** cell proliferation assay, disulfide‐directed β‐hairpin, molecular evolution, NMR spectroscopy, peptide synthesis

## Abstract

Sea stars are an abundant group of marine invertebrates that display remarkably robust regenerative capabilities throughout all life stages. Numerous proteins and peptides have been identified in a proteome study on the coelomic fluid (biofluid) of the common sea star *Asterias rubens*, which appear to be involved with the wound‐healing response in the organism. However, the three‐dimensional structure and function of several of these injury‐responsive peptides, including the peptide KASH2, are yet to be investigated. Here, we show that the KASH2 peptide adopts a disulfide‐directed β‐hairpin fold (DDH). The DDH motif appears to be evolutionarily related to the inhibitor cystine knot motif, which is one of the most widespread disulfide‐rich peptide folds. The DDH motif was originally thought to be restricted to arachnids, but our study suggests that as a result of convergent evolution it could also have originated in sea stars. Although the widely conserved DDH fold has potential cross‐phyla wound‐healing capacity, we have shown that KASH2 does not enhance the proliferation of human fibroblasts, a simple method for wound‐healing re‐epithelialisation screening. Therefore, additional research is necessary to determine the role of KASH2 in the sea stars.

## Graphical abstract

Sea stars have remarkable regenerative capabilities. A peptide, KASH2, identified in the biofluid of the common sea star *Asterias rubens* appears to be involved with the wound‐healing response in the organism. Here, we show the 3D structure of KASH2 adopts a disulfide‐directed β‐hairpin fold, a fold only previously found in arachnids, and does not show wound healing in human fibroblasts.

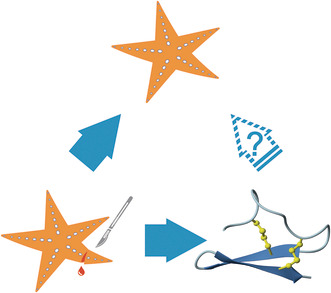

**Abbreviations**
Acm: acetamidomethyl
ACN: acetonitrile
BPP: Bayesian posterior probability
CHCA: α–cyano‐4‐hydroxycinnamic acid
DDH: disulfide‐directed β‐hairpin
DMEM/F12: Dulbecco's modified Eagle medium/nutrient mixture F‐12
DMF: dimethylformamide
FA: formic acid
FBS: fetal bovine serum
Fmoc: fluorenylmethoxycarbonyl
HCTU: *O*‐(1*H*‐6‐chlorobenzotriazole‐1‐yl)‐1,1,3,3‐tetramethyluronium hexafluorophosphate
ICK: inhibitory cystine knot
MALDI‐TOF: matrix‐assisted laser desorption ionisation time‐of‐flight
ML: maximum likelihood
NMR: nuclear magnetic resonance
RP‐HPLC: reversed‐phase high‐performance liquid chromatography
RP‐HPLC/MS: reversed‐phase high‐performance liquid chromatography mass spectrometry
SPPS: solid‐phase peptide synthesis
TFA: trifluoroacetic acid
TIPS: triisopropyl silane
Trt: trityl

Sea stars are invertebrates with exceptional powers of regeneration and the capability to regrow lost limbs [1]. Several molecules have been shown to be involved in regeneration, including the serine protease calpain [2], neurotransmitters (monoamines), neuropeptides (substance P, SALMFamides 1 and 2) and growth‐factor‐like molecules [3], and a recent study suggested that there could be many more [4].

Proteomic analysis of cell‐free coelomic fluid (biofluid) of the common sea star *Asterias rubens* highlighted several proteins that were upregulated in response to injury and, therefore, are potentially involved in defence, cell migration and wound healing [4]. In particular, these studies followed changes that occurred in response to puncture wounds and blood loss. The majority of the upregulated proteins were classified as pattern‐recognition receptors or peptidase inhibitors. However, there were also several uncharacterised proteins upregulated, including the disulfide‐rich proteins KARTESH (KASH1 and KASH2) and CHUPA (CHU1, CHU2 and CHU3) (Fig. 1). These are novel proteins that are expressed with a signal peptide, an acidic region and the mature peptide region containing four‐cysteine residues. Convertase cleavage sites between the acidic region and the cysteine‐rich regions result in the predicted mature peptides for the KARTESH peptides containing 28–30 residues and the CHUPA peptides containing 35–48 residues.

**Fig. 1:**
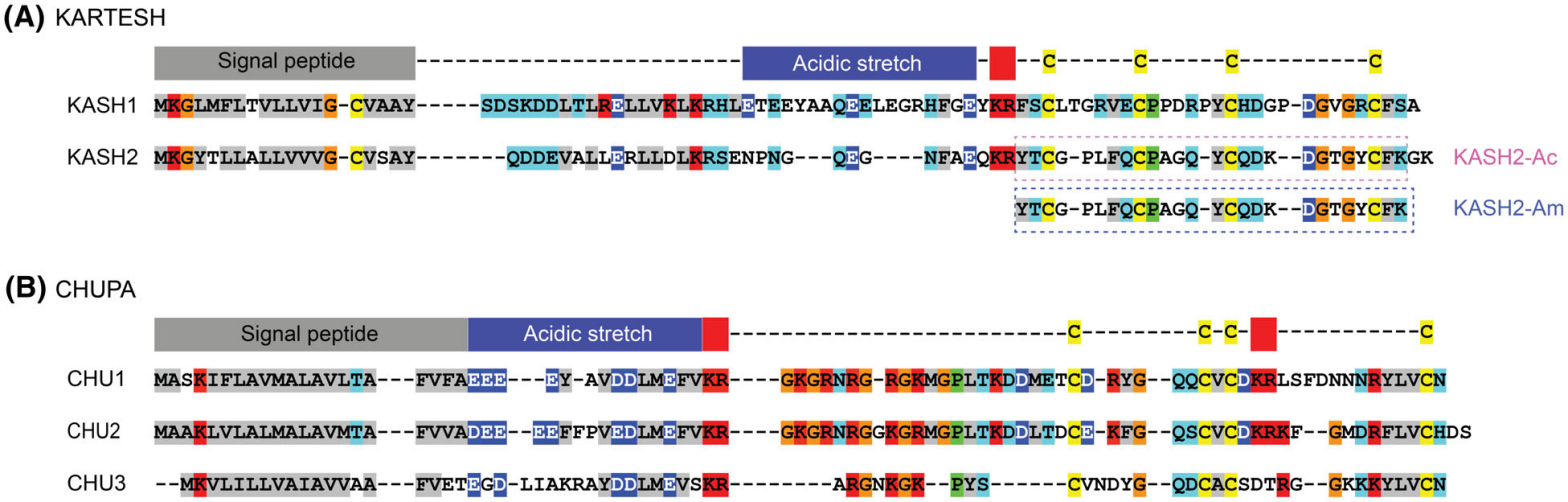
Sequence alignments of peptides from the *Asterias rubens* transcriptome predicted to display wound‐healing properties. (A) KARTESH sequences. (B) CHUPA sequences. Positions of predicted signal peptide, acidic stretch and dibasic convertase cleavage sites are highlighted. The sequences of the synthetic acid and amide versions of KASH2 are also boxed. The mass spectrometry proteomics data and search database associated with these sequences were previously deposited at the ProteomeXchange Consortium via the PRIDE partner repository with the dataset identifier PXD010228. The codes for the sequences are as follows: Kartesh‐2 UniParc·UPI000FECAB46; Kartesh‐1 UniParc·UPI000FECD03F; Chupa‐3 UniParc·UPI000FECB650; Chupa‐2 UniParc·UPI000EB785BA; Chupa‐1 UniParc·UPI000EB634EE.

Several homologues of the KARTESH and CHUPA proteins are present in other echinoderms, but not in any other phyla. The cysteine residues are conserved throughout the predicted mature peptides, but there is variation in the residue type and the number of residues in the inter‐cysteine loops. This phenomenon of families of peptides with inter‐cysteine loop sequence variations combined with conservation of the cysteine residues is prevalent throughout nature, including in the venom peptides of cone snails, spiders and sea anemones, with the former containing numerous peptides with four‐cysteine residues [5, 6, 7, 8, 9, 10, 11, 12, 13].

The upregulation of the uncharacterised sea star proteins in response to injury warrants further investigation. In particular, the determination of the three‐dimensional structures might provide insight into their function and evolution. Here, we have determined the three‐dimensional structure of KASH2 using NMR spectroscopy. Two constructs of the peptides were studied: one with the predicted sequence with a C‐terminal acid (KASH2‐Ac) and the other with a C‐terminal amide (KASH2‐Am), given the conserved amidation signal (Xaa‐Gly‐Lys/Arg) at the C terminus. The sequences of the KASH2 peptides are given in Fig. 1. Intriguingly, we found that the three‐dimensional solution structure contains an ancestral protein fold termed the disulfide‐directed β‐hairpin (DDH), initially defined by Wang *et al*. [12], and previously thought to be restricted to arachnids. As we have previously seen cross‐phyla wound‐healing activity from cysteine‐rich peptides [14, 15, 16], we explored whether the potential for therapeutic human wound‐healing properties is associated with this DDH‐fold peptide, similar to what we have observed for granulin‐derived peptides from the human parasitic liver fluke *Opisthorchis viverrini*, with a human fibroblast proliferation assay [14, 15, 17, 18]. However, neither the acid nor amide versions of KASH2 showed any effect on cell proliferation, suggesting that it might be phylum‐specific or it might not be directly related to wound healing in the sea star.

## Materials and methods

### Peptide synthesis and purification

The KASH2 acid form, KASH2‐Ac and CHUPA1 peptides were synthesised by manual solid‐phase peptide synthesis (SPPS) using Fmoc‐SPPS chemistry and were assembled on 2‐chlorotrityl chloride resin (Auspep, Tullamarine, VIC, Australia). Amino acids were activated using *O*‐(1*H*‐6‐Chlorobenzotriazole‐1‐yl)‐1,1,3,3‐tetramethyluronium hexafluorophosphate (HCTU; Peptides International, Louisville, KY, USA) in peptide grade dimethylformamide (DMF; Auspep). Peptide was cleaved from the resin using a mixture of 95% trifluoroacetic acid (TFA; Auspep)/2.5% triisopropyl silane (TIPS; Sigma‐Aldrich, St. Louis, MO, USA)/2.5% H_2_O at room temperature for 2 h [14, 16], and the TFA was removed by evaporation. Cleaved peptide was precipitated with ice‐cold diethyl ether. Diethyl ether was removed by filtration, and the precipitated peptide was dissolved in 50% acetonitrile/50% H_2_O/0.1% TFA (v/v/v) and subsequently lyophilised. The resulting crude peptide was purified with reversed‐phase high‐performance liquid chromatography (RP‐HPLC) (Agilent 1260 Infinity; Agilent Technologies, Mulgrave, VIC, Australia) on a C_18_ preparative column (Phenomenex Jupiter 10 μm, C_18_, 300 Å, 250 mm × 21.2 mm) using an appropriate gradient of solvents A (0.05% TFA in water) and B (0.045% TFA, 90% acetonitrile in water). UV absorbance was monitored at 214 and 280 nm. The purity of the peptides was assessed using analytical RP‐HPLC on a C_18_ analytical column (Agilent Eclipse Plus C_18_, 3.5 μm, 4.6 × 100 mm or Phenomenex Aeris 3.5 μm PEPTIDE XB‐C18, 100 Å, 150 × 2.1 mm) using a gradient of 0–50% solvent B in 50 min, 50–90% solvent B in 5 min, 90–0% solvent B in 5 min at 1 mL·min^−1^ or a gradient of 0–60% solvent B in 60 min at 0.25 mL·min^−1^, respectively. The KASH2 peptide with a C‐terminal amide, KASH2‐Am, was purchased from GenScript (GenScript, Piscataway, NJ, USA), with the cysteine residues in the reduced form.

### Disulfide formation

Both the C‐terminal acid and amide versions of KASH2 were oxidised by dissolving lyophilised, purified peptide in 100 mm ammonium bicarbonate (pH 8.2) for 24 h at room temperature. Oxidised peptides were acidified, filtered and purified using RP‐HPLC on a C_18_ preparative column (Phenomenex Jupiter 10 μm C_18_ 300 Å, 250 × 21.2 mm). The purity of the peptides was assessed using analytical RP‐HPLC on the same C_18_ analytical column and gradient as above. The peptide masses were analysed by matrix‐assisted laser desorption ionisation time‐of‐flight (MALDI‐TOF) mass spectrometry using a 5800 MALDI TOF/TOF spectrometer (SCIEX, Framingham, MA, USA). Samples (0.5 μL) were spotted onto a 384‐well stainless‐steel target plate with 0.5 μL of α–cyano‐4‐hydroxycinnamic acid (CHCA; Sigma‐Aldrich, St. Louis, MO, USA) matrix. Analysis used reflector positive mode with a scan range of *m/z* 800–4500, averaged over 2000 laser shots. Samples were also analysed using RP‐HPLC/mass spectrometry (RP‐HPLC/MS) on a Shimadzu LCMS‐2020 mass spectrometer combined with a Shimadzu Prominence HPLC system (Shimadzu, Kyoto, Japan). Samples were injected onto a RP‐HPLC column (Phenomenex Aeris 3.6 μm PEPTIDE XB‐C18 100 Å, 2.1 × 150 mm; Phenomenex, Torrance, CA, USA) at 30 °C and eluted using a 1% gradient of RP‐HPLC/MS solvent B (90% acetonitrile [ACN; OPTIMA LCMS grade, Thermo Fisher Scientific, Scoresby, Vic., Australia]/0.09% formic acid [FA; Sigma‐Aldrich]/water) in RP‐HPLC/MS solvent A (0.1% FA) from 0% to 60% LC/MS solvent B over 60 min at a flow rate of 0.250 mL·min^−1^. UV absorbance was monitored at 214 and 280 nm, and mass spectra were collected in positive ion mode with a scan range of *m/z* 250–2000, detector voltage of 1.15 kV, nebulising gas flow of 1.5 L·min^−1^ and drying gas flow of 3.0 L·min^−1^. Shimadzu labsolutions v5.96 software (Shimadzu) was used to collect and analyse data.

Although the native connectivity of KASH2 is not known, to determine the disulfide connectivity present in the major isomer in the one‐step oxidative folding of the C‐terminal acid version, selective protection of the cysteine residues was carried out. Side‐chain protection with acetamidomethyl (Acm) groups was used for Cys II and IV, and trityl (Trt) protecting groups were used for Cys I and Cys III in the synthesis. Following TFA cleavage and deprotection in the presence of scavenger (TIPS), the disulfide bond between Cys I and Cys III was formed by air oxidation in ammonium bicarbonate (pH 8.2) for 24 h, and the peptide was purified using RP‐HPLC (as above). The second disulfide bond was formed following removal of the Acm protecting group from Cys II and Cys IV. Cys‐Acm peptide was dissolved in 50% aqueous acetic acid (~ 0.5 mg·mL^−1^), 1 m HCl (0.1 mL·mg^−1^ of peptide) was then added, followed immediately by I_2_ in 100% aqueous acetic acid until the solution was yellow. The flask was flushed with nitrogen and sealed. The reaction was left at room temperature for 2 h and then quenched by adding 1 m ascorbic acid solution dropwise until the solution was colourless. Finally, the quenched reaction was diluted with RP‐HPLC solvent A until the percentage of acetic acid was ~ 10%. Oxidised peptide was purified using a C_12_ semi‐preparative column (Phenomenex Jupiter Proteo C_12_, 4 μm, 90 Å, 250 × 10.0 mm) or a C_18_ semi‐preparative column (Phenomenex Aeris 5 μm PEPTIDE XB‐C18, 100 Å, 250 × 10.0 mm). The purity of the peptides was assessed using analytical RP‐HPLC on a C_18_ analytical column (Agilent Eclipse Plus C_18_, 3.5 μm, 4.6 × 100 mm or Phenomenex Aeris 3.5 μm PEPTIDE XB‐C18, 100 Å, 150 × 2.1 mm) using a gradient of 0–50% solvent B in 50 min, 50–90% solvent B in 5 min, 90–0% solvent B in 5 min at 1 mL·min^−1^ or a gradient of 0–60% solvent B in 60 min at 0.25 mL·min^−1^, respectively.

### NMR spectroscopy and structure determination

NMR spectra were recorded at 290 K on Bruker Avance III 600 MHz spectrometer (Bruker, Karlsruhe, Germany) equipped with a TCI cryoprobe. Samples were prepared from lyophilised peptides at concentrations of approximately 0.2 mm in 90% H_2_O/10% D_2_O (D_2_O; Cambridge Isotope Laboratories, Woburn, MA, USA). Two‐dimensional spectra including ^1^H–^1^H TOCSY, ^1^H–^1^H NOESY, ^1^H–^1^H DQF‐COSY, ^1^H–^15^N HSQC and ^1^H–^13^C HSQC were used for assignment. TOCSY and NOESY spectra were collected using mixing times of 80 and 200 ms, respectively. A series of one‐dimensional proton and TOCSY spectra were recorded following dissolution of the peptides in 100% D_2_O to monitor the rate of exchange of the amide protons. All spectra were analysed using topspin (Bruker, Billerica, MA, USA) and assigned using ccpnmr based on the approach described by Wüthrich [19, 20]. The αH secondary chemical shifts were determined by subtracting random coil ^1^H NMR chemical shifts from the experimental αH chemical shifts [21]. The 2D NOESY spectra were assigned and an ensemble of structures calculated using the program cyana [22]. A total of 100 initial structures were calculated using the cyana program. Torsion‐angle restraints predicted using talos‐n were used in the structure calculations [23]. The disulfide‐bond connectivities were included in the final calculations of KASH2‐Ac and KASH2‐Am. A final ensemble of the 20 lowest energy structures with no distance violations > 0.5 Å or angle violations > 4° was prepared. Structures were visualised using molmol [24]. The structures and chemical shifts of KASH2‐Ac (PDB ID: 8V2M, BMRB ID: 31128) and KASH2‐Am (PDB ID: 8V2U, BMRB ID: 31129) have been deposited into the Protein Data Bank and the Biological Magnetic Resonance Data Bank.

### Sequence retrieval and alignment

A DDH nucleotide dataset was assembled using sequences from the NCBI‐NR database (https://www.ncbi.nlm.nih.gov/). KASH2, a peptide from the common sea star (*A. rubens*) and associated with wound‐healing and adopting the DDH fold, was used as query sequences for blast searches (https://blast.ncbi.nlm.nih.gov/BlastAlign.cgi). Sequences obtained were manually curated before alignment. Translated sequences were aligned in mega7 using the muscle algorithm [25, 26]. Universally conserved cysteines conforming to the DDH motif were used as guides to refine the alignment. Regions with gaps in the alignment of > 50% were trimmed prior to phylogenetic analyses.

### Phylogenetic analysis

The evolutionary relationship between DDH sequences was determined by subjecting the nucleotide dataset to Bayesian and maximum likelihood (ML) analyses. The MPI version of mrbayes 3.2.6 [27, 28] was implemented to perform Bayesian inferences. Analyses were run for a minimum of two hundred million generations using 12 Markov chains across four runs, sampling every 100th tree. Twenty‐five per cent of the sampled trees were discarded as burn‐in. The log‐likelihood score for each of the saved trees was plotted against the number of generations to assess if the log‐likelihood scores of the analyses had reached asymptote. Bayesian posterior probability (BPP) was used to evaluate the branch node support. ML analyses were performed using iq‐tree v1.6.12 [29, 30] with an Edge‐proportional partition model coupled with modelfinder for tree reconstruction using the best‐fit partitioning scheme. Bootstrap was used to evaluate branch node support and analyses were run for 100 bootstrap replicates. All phylogenetic trees were midpoint rooted and were visualised using figtree v1.4.4 (http://tree.bio.ed.ac.uk/software/figtree/).

### Human skin normal fibroblast cells

The human skin normal fibroblast cell line 1BR.3.GN was obtained from a European Collection of Authenticated Cell Cultures (ECACC, Porton Down, UK). The 1BR.3.GN cells were grown and maintained in complete media: Dulbecco's modified Eagle medium/Nutrient Mixture F‐12 (DMEM/F12) with GlutaMAX (Life Technologies, Melbourne, Vic., Australia) containing 1 × antibiotic/antimycotic and 1 × GlutaMAX, supplemented with 10% fetal bovine serum (FBS) (Gibco, Glasgow, UK) at 37 °C and 5% CO_2_. Cell proliferation assays were performed with low‐nutrient media: DMEM/F12 low‐nutrient media supplemented with 0.5% FBS and 1 × antibiotic/antimycotic.

### The real‐time xCELLigence cell proliferation assay

Cells were seeded at 5000 cells per well in 150 μL of complete media in E‐plates (ACEA Biosciences, San Diego, CA, USA) and grown overnight while monitoring with an xCELLigence SP system (ACEA Biosciences), which monitors cellular events in real time by measuring electrical impedance across gold microelectrodes integrated into the base of tissue culture plates. Cells were washed three times with low‐nutrient media prior to the addition of 150 μL of low‐nutrient media and incubated for a minimum of 6 h before further treatment. Treatments were prepared at 8.5 × concentration and added to each of six replicate wells in 20 μL, for a final 1 × concentration in a total volume of 170 μL. The xCELLigence system recorded cell indices at intervals of 1 h for 5–6 days following treatment. Readings for the cell index were normalised before treatment, and cell proliferation ratios characterised the relative numbers of cells compared to control cells. GRN_P4A_ was used as a positive control in the cell proliferation assay, and a scrambled version of this peptide (ALPYSPSGHGGRSSGTDSVSYQTR), referred to as Gsp, was used as a negative control. GRN_P4A_ is a liver fluke‐derived granulin peptide that significantly accelerates proliferation of fibroblasts [15]. Day 4 cell proliferation rates were compared between treatment and control wells, and a one‐way ANOVA test was used for multiple comparisons with Holm–Sidak's correction, using graphpad prism 9.0 (GraphPad Software, San Diego, CA, USA).

## Results

Although several peptides and proteins have been shown to be upregulated in *A. rubens* in response to injury [4], the current study focussed on KASH2 because marine‐derived cysteine‐rich peptides have been shown to have potential in drug design and structural studies could provide insight into molecular evolution. Furthermore, detailed studies were carried out on KASH2 because our preliminary results on the CHUPA3 peptide indicated that the peptide did not fold into a well‐defined structure *in vitro* and therefore would be difficult to discern the most likely native fold.

### Peptide synthesis

**Fig. 2:**
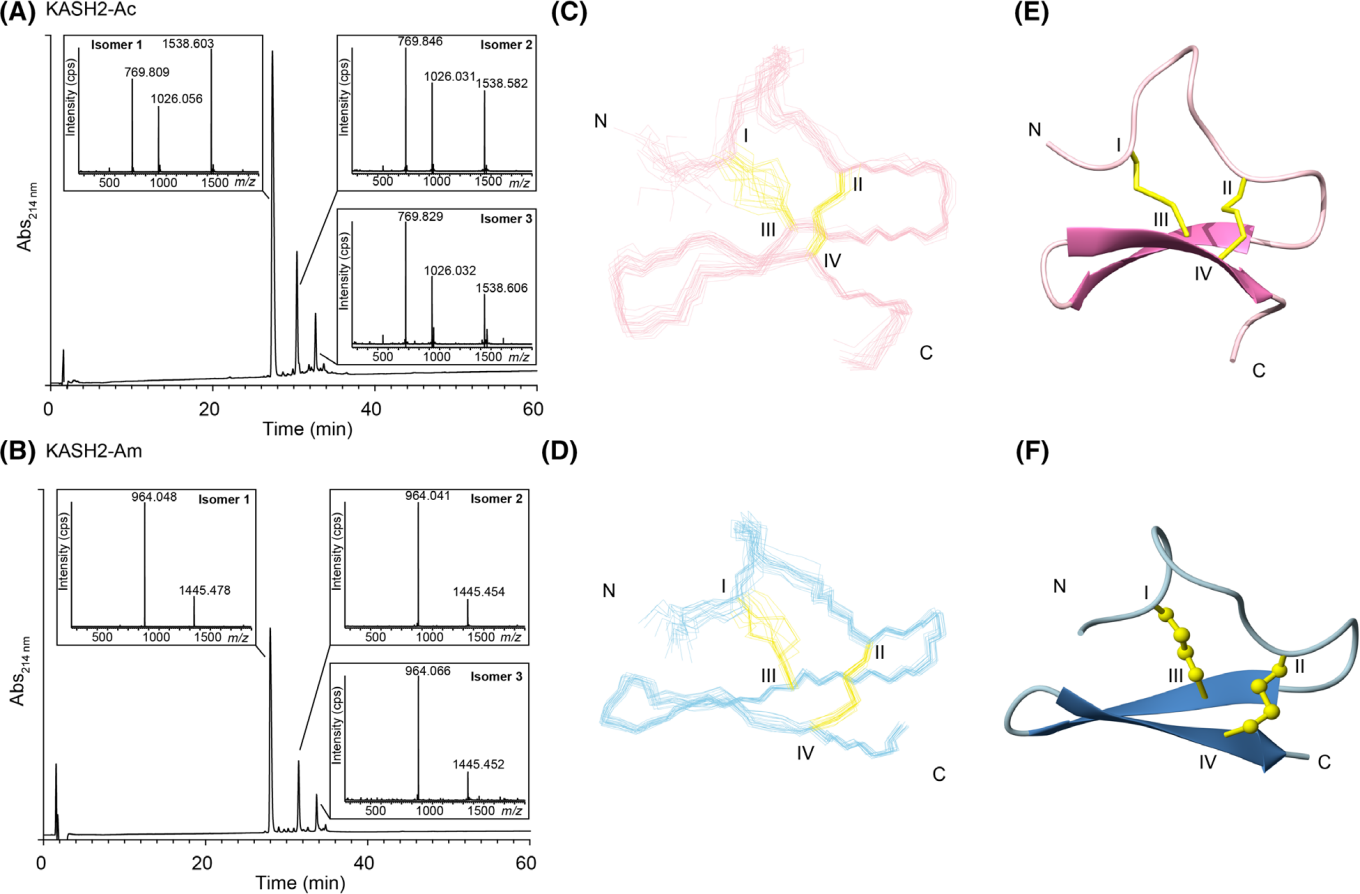
Oxidative folding of KASH2. RP‐HPLC/MS analysis of the oxidation reactions of (A) KASH2‐Ac and (B) KASH2‐Am. Three main peaks were observed for both peptides, with the first peak being the predominant form. The masses for each of the peaks are given as insets to the figure and show the same mass for each of the isomers within a reaction, indicating they represent disulfide‐bond isomers. (C) The three‐dimensional structures of the 20 lowest energy structures of KASH2‐Ac (PDB: 8V2M), and (D) KASH2‐Am (PDB: 8V2U), determined based on NMR data. (E) Ribbon format of KASH2‐Ac (PDB: 8V2M), and (F) KASH2‐Am (PDB: 8V2U). Cysteines are numbered with Roman numerals.

The sequence corresponding to the expected KASH2 and CHUPA3 mature peptides (Fig. 1) were synthesised using Fmoc‐SPPS. It is possible that KASH2 has an amidated C terminus based on the conserved sequence corresponding to an amidation signal, and consequently, the peptide was also synthesised with a C‐terminal amide (this peptide has 26 residues rather than 28 for the acid peptide). This is in contrast to KASH1, which appears to have a C‐terminal acid based on the absence of the conserved motif associated with amidation. The crude peptides were purified using RP‐HPLC, and mass analysis was carried out using MALDI mass spectrometry. Oxidative folding was carried out in a single‐step oxidation reaction using ammonium bicarbonate. The oxidation of CHUPA‐3 did not produce well‐resolved isomers and further study was not carried out on this peptide. By contrast, similar results were observed for both KASH2‐Ac and KASH2‐Am, and the analytical RP‐HPLC trace of the oxidation reactions contained three main peaks at different retention times but the same mass (confirmed by RP‐HPLC/MS analysis), indicating the presence of three isomers with different disulfide‐bond connectivities, as shown in Fig. 2A,B. The earliest eluting peak, subsequently referred to as isomer 1, was the major isomer, most likely representing the lowest energy/most stable isomer. For both KASH2‐Ac and KASH2‐Am, all three isomers were subsequently purified using RP‐HPLC for further analysis.

### NMR spectroscopy and structure determination

The structures of the KASH2 isomers were analysed using NMR spectroscopy. The pH of the NMR sample solution was ~ 4.5. Spectra also recorded at higher pH (~ 5.5) had greater amide exchange (Fig. S1), which prevented the assignment of several resonances and were not involved in further analysis. For both peptides, the first peak (isomer 1) in the HPLC trace had significant dispersion in the amide region indicative of a well‐structured peptide (Fig. S2), in contrast to the other isomers, which had significant overlap in this region. The three‐dimensional structure of isomer 1 was determined for both KASH2‐Ac and KASH2‐Am using torsion‐angle dynamics in the cyana program (see Figs S3–S12 for 2D NMR data). The major element of the secondary structure was a β‐hairpin braced by two disulfide bonds. The proline residues appear to be in trans conformation based on the α‐δ NOEs, and the ^13^C chemical shifts for the beta and gamma carbon atoms (Fig. S13), which are consistent with trans isomers based on the study by Schubert *et al*. [31]. These ^13^C chemical shifts for isomers 1 of KASH2‐Ac and KASH2‐Am are given in Table S1. The preliminary structures and slow exchange data were used to infer the hydrogen bonds (Table S2), which were then included in the structure refinements.

Structures were initially calculated with no disulfide‐bond restraints, and based on the inter‐cysteine distances measured in molmol, the most likely disulfide bond was between Cys II to Cys IV. Consequently, the most likely connectivity involved Cys I–Cys III, Cys II–Cys IV. Structures calculated with this connectivity are shown in Fig. 2C–F. The refinement statistics for the final structures are provided in Table S3. Analysis of the three‐dimensional structure of KASH2 indicates that it contains the DDH motif based on the disulfide connectivity with respect to the β‐hairpin [12]. Although both forms of the peptide have similar structures, the peptide containing the C‐terminal amide has more NOE restraints and consequently provided a more refined structure. Analysis of the NOE restraints between the two structures indicated that the increased number of NOEs occurs throughout the molecule, but in particular involves residues 16 and 18. Therefore, it appears that the C‐terminal amide is having an influence on the overall structure, but it should be noted that this peptide is shorter than the acid version, and this might also be having an impact on the structural definition. A more well‐defined structure is possibly linked to greater stability, suggesting that the C‐terminal amide is present in the native peptide, but this is speculation at this stage.

### KASH2 synthesis using selective protection of the cysteine residues

To confirm the disulfide connectivity present in isomer 1 of KASH2, selective protection of the cysteine residues was used to direct the folding to form the predicted disulfide connectivity (Cys I–Cys III and Cys II–Cys IV) (Fig. S14). The experiments were done on the C‐terminal acid form. In the synthesis of the peptide, side‐chain protection was used for Cys II and IV (Acm) and protecting groups were used for Cys I and Cys III (Trt). Following deprotection, the disulfide bond between Cys I and Cys III was formed by air oxidation and the second disulfide bond was formed following removal of the protecting groups from Cys II and Cys IV. The fully oxidised peptide was subsequently purified using RP‐HPLC and shown to have a similar retention time and similar NMR spectra to KASH2‐Ac isomer 1 (Fig. S2). A comparison of the chemical shifts is given in Fig. S15.

### Phylogenetic analyses of a DDH peptide identified from the common sea star *A. rubens*

The DDH is a part of the disulfide‐rich peptide class of proteins, which includes other members such as the inhibitory cystine knot (ICK) [7, 32, 33] and the disulfide‐stabilised antiparallel β‐hairpin stack [7]. The various disulfide linkages in these peptides play a crucial role in determining their native conformation and biological function. Examples of these different classes of disulfide‐rich peptides have been found in arachnid toxins [33, 34] and have also been detected in other organisms including plants and fungi [35]. However, KASH2 is the first peptide to contain the DDH fold reported from outside the arachnid lineage and warrants further investigation to understand the evolutionary origin and diversification of disulfide‐rich peptides.

In our in‐depth searches against various sea star genomes and other sequences from public repositories, we could not retrieve homologues for KASH2. Very limited positive hits in certain sea star genomes turned out to be characterised by very poor coverage, and therefore, we could not use these in our analyses. The only exceptions were the sequences that we retrieved from the *A. rubens* genome.

**Fig. 3:**
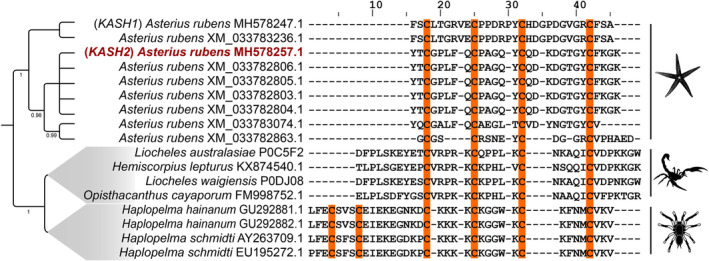
The Bayesian phylogeny and sequence alignment of DDH peptides from *Asterias rubens* and Arachnida. This figure depicts the representative Bayesian relationship between sea star (*A. rubens*) and arachnid DDH sequences along with, sequence alignment of the mature DDH peptides from *A. rubens* and Arachnida. Here, the KASH2 peptide from *A. rubens* is shown in red, while the cysteine residues involved in the formation of the DDH fold are highlighted in orange.

To understand their evolutionary history, we performed phylogenetic reconstructions of all known peptides from arachnids and sequences retrieved from *A. rubens* containing the DDH motif. We observed that the DDH‐containing sequences identified from *A. rubens* form a distinct clade from the known arachnid DDH sequences (Figs S16 and S17, with highlights shown in Fig. 3). When compared and aligned with the known DDH sequences from arachnids, we found that the sequences from *A. rubens* are relatively short in length, and share very little sequence similarity with their arachnid DDH counterparts, except for the four corresponding cysteine residues. The three‐dimensional structure of KASH2 is consistent with the DDH motif, as it adopts the typical disulfide bridging [C1–C3] [C2–C4] pattern, similar to U_1_‐LITX‐Lw1a identified from the scorpion *Liocheles waigiensis* (Fig. 3).

Based on our phylogenetic analyses and the sequence comparisons of DDH‐containing peptides, we conclude that the presence of DDH motifs found in the European or common sea star is likely a result of convergent evolution (Figs S16 and S17).

### Cell proliferation monitoring in real time using xCELLigence

**Fig. 4:**
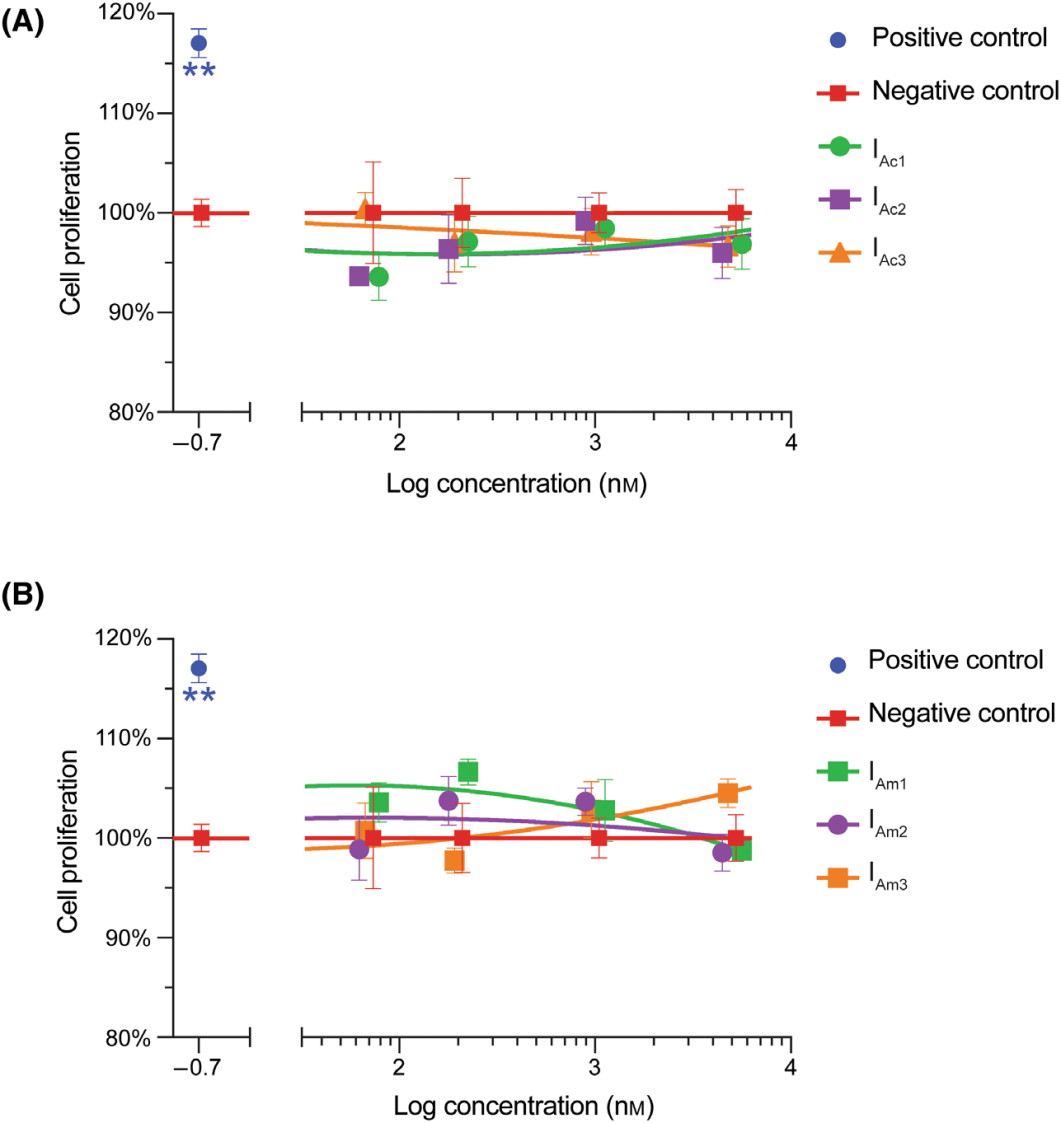
The influence of KASH2 peptides on the cell proliferation of human fibroblasts. Human fibroblast cell line was incubated with the (A) three KASH2‐Ac and (B) KASH2‐Am isomers and analysed using an xCELLigence system over 120 h. Cell proliferation is plotted relative to Gsp (scrambled granulin) controls with five replicates and pooled from four separate experiments (*n* = 4). Data as analysed with a one‐way ANOVA with Holm–Sidak multiple comparison against Gsp control: **P < 0.01, unlabelled = not significant.

The effect of both KASH2‐Ac and KASH2‐Am peptides on the stimulation of an *in vitro* cell proliferation assay of 1BR.3.GN fibroblasts was carried out using an xCELLigence system at the range of concentrations (0.32–1000 nm) at peak cell index (~ 120 h/5 days). The KASH2 peptides did not show significant cell proliferation on human fibroblast cell growth over the concentration range tested. The positive control, GRN_P4A_, showed approximately 115% cell proliferation relative to the negative control as shown in Fig. 4 and was consistent with previous results [15].

## Discussion

Several proteins from *A. rubens* have recently been shown to be upregulated in response to injury, including proteins with no known function or structural information [4]. Determination of the three‐dimensional structure of one of the mature peptides predicted from the identified precursor proteins has provided insight into the molecular evolution of a disulfide‐rich structural motif.

The synthetic version of KASH2 with a C‐terminal acid produced three isomers during the oxidative folding reaction, which most likely correspond to the three possible disulfide connectivities for a four‐cysteine containing peptide. For small disulfide‐rich peptides, it is quite common for the earliest eluting peak on RP‐HPLC to correspond to the natively folded peptide. This appears to be the case for KASH2, as the earliest eluting peak was the major form and displays a well‐defined structure. Preliminary structural analyses of this isomer suggested that the disulfide connectivity was Cys I–Cys III and Cys II–Cys IV, and this was subsequently confirmed by selective protection/deprotection oxidation of the cysteine residues.

Similar results were observed in the oxidative folding of the KASH2 peptide with the C‐terminal amide with two less residues than the C‐terminal acid form. The structures of the major isomers of KASH2‐Ac and KASH2‐Am peptides were very similar in overall fold, but the amidated version was more well‐defined than the acid version. Peptides with amidated C‐termini are widespread throughout nature, particularly in disulfide‐rich peptides from cone snail venom. C‐terminal amidation of an α‐conotoxin has previously been shown to stabilise the secondary structure [36], similar to the results observed in the current study. For the α‐conotoxin, it was suggested that this amide stabilisation played a role in receptor binding rather than interacting directly with the binding site.

**Fig. 5:**
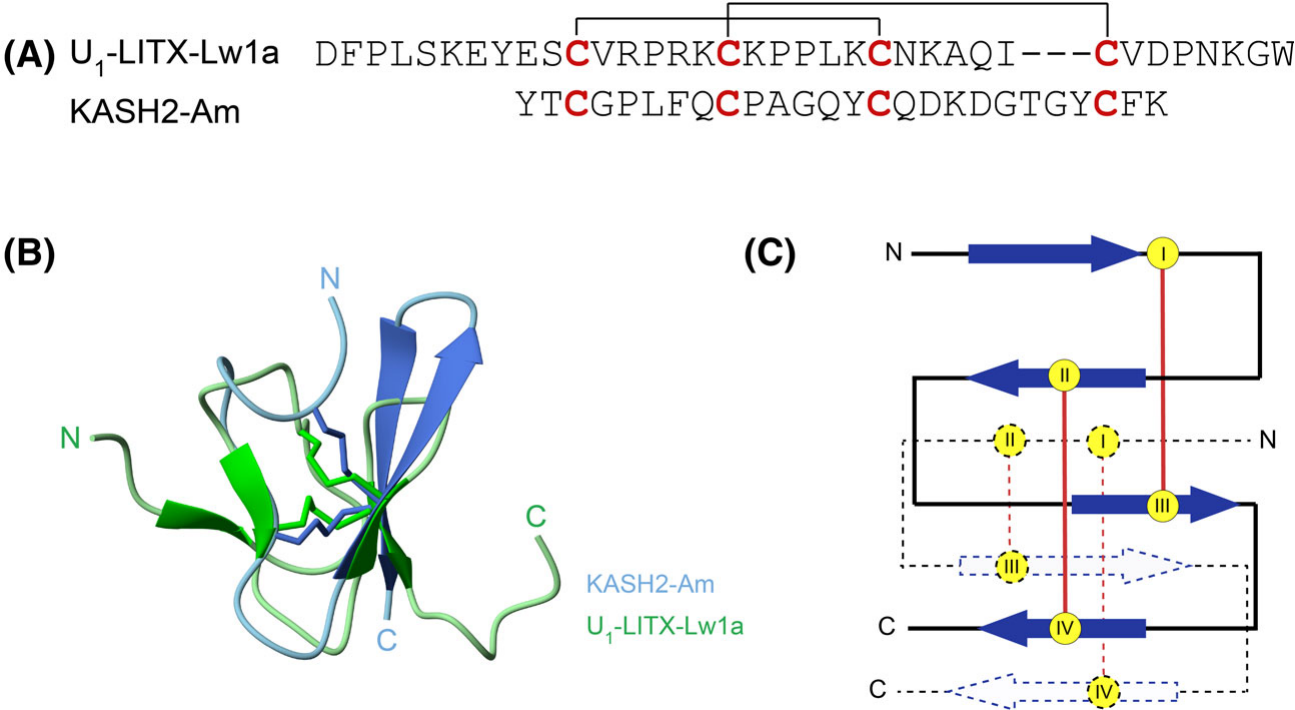
Conserved structural fold between sea star *Asterias rubens* and scorpion *Liocheles waigiensis* peptides. (A) Alignment of the sequences with cysteine connectivities shown with lines. (B) Overlay of the three‐dimensional structures of U_1_‐LITX‐Lw1a PDB ID: 2KYJ and KASH2‐Am, superimposed over the cysteine residues. (C) Graphical representation of the DDH motif, with the β‐sheets shown as arrows and cysteine residues labelled with Roman numerals. The KASH2 DDH motif is shown using a dashed line.

Analysis of the three‐dimensional structure of KASH2 with the VAST [37] structural similarity program indicated that KASH2 does not have significant structural similarity to any characterised peptides. However, many toxin peptides are known to contain disulfide‐rich motifs, and we noticed that KASH2 has the same disulfide connectivity [I–III, II–IV] and similar fold to the scorpion toxin U_1_‐LITX‐Lw1a [10] as shown in Fig. 5. U_1_‐LITX‐Lw1a has more residues than KASH2 and contains an additional β‐hairpin, but the RMSD between residues 17–23, 28–30 in U1‐LITX‐Lw1a, and 9–15, 23–25 in KASH2 is 0.532 (Å), highlighting the similarity between the peptides. The single‐domain DDH fold was first reported in the native toxin peptide, J‐ACTX‐Hv1c, from the Australian funnel‐web spider [8, 12] and has subsequently been shown in U_1_‐LITX‐Lw1a [10].

In terms of structural evolution of peptides such as KASH2, the Arachnida and Asteroidea have been phylogenetically separated for nearly 560 million years. It is likely that the two lineages adopted the DDH scaffold from the same/related cysteine‐rich scaffold subsequent to their divergence from the most recent common ancestor. Further analyses, such as gene synteny and the identification of similar disulfide‐rich peptides along the phylogenetic tree leading to Arachnida and Asteroidea, would help in delineating the molecular phylogeny more precisely. However, with the existing evidence, the independent origin of DDH in sea star and arachnids seems to be the most parsimonious explanation.

There has been speculation regarding the evolution of the ICK motif, with one hypothesis being that the DDH motif is an ancestral fold of the ICK motif [12]. However, a subsequent study suggested that although the structural data of U_1_‐LITX‐Lw1a support an ancestral link between the ICK and DDH motifs, molecular evolution data and the apparent restriction of single‐domain DDH peptides to arachnids suggested that DDH is a derived ICK and that this derivation has occurred on multiple occasions [33]. Consequently, our finding of a single‐domain DDH peptide in sea stars might have implications for future studies on the evolution of the ICK fold.

Sea stars have been shown to have remarkable abilities to regenerate tissue [38, 39]. Our team explored evolutionary conserved structural elements across phyla that have human wound‐healing potential. As part of our exploration of cross‐phyla structure–function relationships, it was of interest to investigate the biological activity of KASH2 in terms of human cell proliferation. The lack of activity of KASH2 on the proliferation of human fibroblast rules out the cross‐phyla structural wound‐healing aspect. It is possible that KASH2 is either not directly related to wound healing in the sea star or is part of a proteome that activates receptors not involved in human wound healing. In order to further explore the potential bioactivities associated with this peptide, collection of coelomic fluid (containing coelomocytes) from live animals and measuring cell growth *in vitro* immediately after collection and after addition of KASH2 might be considered as a preliminary screen. However, detailed investigations would be required to understand the actual functions of KASH2 in wound healing in *A. rubens*.

## Conclusions

Overall, we have identified the first DDH structural motif peptide characterised from the sea star *A. rubens*. We have significantly expanded the sequence diversity known to adopt this fold by placing DDH in both the Echinodermata and Arthropoda phyla. Although KASH2 was upregulated in response to injury, we show here that it did not have any effect on the cell proliferation of human fibroblasts. There are numerous other roles it might play in injury in *A. rubens*, which remain to be determined. Disulfide‐rich scaffolds, such as the DDH fold, have previously been shown to be useful as scaffolds in drug design and agricultural applications [9, 40, 41, 42], and the KASH2 peptide might add to the library of peptides available for these applications.

## Conflict of interest

The authors declare no conflict of interest.

## Author contributions

RT and NLD contributed to conceptualisation. RT, NLD, DTW and MJS contributed to methodology. RT, NLD, DTW, JLQ, MJS, CAS, NYS and KS contributed to investigation. RT and NLD contributed to visualisation. NLD, DTW, MJS and MJL contributed to supervision. RT and NLD contributed to writing—original draft. RT, NLD, DTW, MJS, AL, CAS, NYS, KS and MJL contributed to writing—review and editing.

## Supporting information


**Fig. S1.** KASH2‐Ac at pH 5.5 ^1^H NMR (600 MHz, 90% H_2_O/10% D_2_O v/v) spectra.
**Fig. S2.** KASH2‐Ac, ‐Am ^1^H NMR (600 MHz, 90% H_2_O/10% D_2_O v/v) spectrum.
**Fig. S3.** KASH2‐Ac isomer 1 ^1^H–^1^H TOCSY NMR (600 MHz, 90% H_2_O/10% D_2_O v/v) spectrum.
**Fig. S4.** KASH2‐Ac isomer 1 ^1^H–^1^H NOESY NMR (600 MHz, 90% H_2_O/10% D_2_O v/v) spectrum.
**Fig. S5.** KASH2‐Ac isomer 1 ^1^H–^1^H COSY NMR (600 MHz, 90% H_2_O/10% D_2_O v/v) spectrum.
**Fig. S6.** KASH2‐Ac isomer 1 ^1^H–^15^N HSQC NMR (600 MHz, 90% H_2_O/10% D_2_O v/v) spectrum.
**Fig. S7.** KASH2‐Ac isomer 1 ^1^H–^13^C HSQC NMR (600 MHz, 90% H_2_O/10% D_2_O v/v) spectrum.
**Fig. S8.** KASH2‐Am isomer 1 ^1^H–^1^H TOCSY NMR (600 MHz, 90% H_2_O/10% D_2_O v/v) spectrum.
**Fig. S9.** KASH2‐Am isomer 1 ^1^H–^1^H NOESY NMR (600 MHz, 90% H_2_O/10% D_2_O v/v) spectrum.
**Fig. S10.** KASH2‐Am isomer 1 ^1^H–^1^H COSY NMR (600 MHz, 90% H_2_O/10% D_2_O v/v) spectrum.
**Fig. S11.** KASH2‐Am isomer 1 ^1^H–^15^N HSQC NMR (600 MHz, 90% H_2_O/10% D_2_O v/v) spectrum.
**Fig. S12.** KASH2‐Am isomer 1 ^1^H–^13^C HSQC NMR (600 MHz, 90% H_2_O/10% D_2_O v/v) spectrum.
**Fig. S13.** ^1^H–^1^H NOESY NMR spectra (600 MHz, 90% H_2_O/10% D_2_O v/v) of KASH2 peptides showing the NOEs consistent with the trans conformation.
**Fig. S14.** Characterisation by RP‐HPLC and RP‐HPLC/MS of KASH2‐Ac produced with selective protection of the cysteine residues.
**Fig. S15.** αH Secondary chemical shifts for KASH2 peptides.
**Fig. S16.** A Bayesian phylogeny of DDH motifs.
**Fig. S17.** Maximum likelihood phylogeny of the DDH motif.
**Table S1.** Analysis of the backbone conformation of proline residues in KASH2 isomers.
**Table S2.** Hydrogen bond restraints for KASH2.
**Table S3.** Structural statistics for KASH2.

## Data Availability

All data are available in the main text or the Supporting Information. The coordinates of the structures of the KASH2‐Ac and KASH2‐Am peptides are available from the Protein Data Bank and the Biological Magnetic Resonance Data Bank with accession numbers (PDB ID: 8V2M, BMRB ID: 31128) and (PDB ID: 8V2U, BMRB ID: 31129), respectively.
