# Supplementary material for: Structural analysis of an Asterias rubens peptide indicates the presence of a disulfide‐directed β‐hairpin fold
Source: FEBS Open Bio. 2024 Nov 19;15(3):415–26. doi: 10.1002/2211-5463.13931 (PMC11891777; doi:10.1002/2211-5463.13931)
Supplement: Supplementary file 1 — Fig. S1. KASH2‐Ac at pH 5.5 1H NMR (600 MHz, 90% H2O/10% D2O v/v) spectra. Fig. S2. KASH2‐Ac, ‐Am 1H NMR (600 MHz, 90% H2O/10% D2O v/v) spectrum. Fig. S3. KASH2‐Ac isomer 1 1H–1H TOCSY NMR (600 MHz, 90% H2O/10% D2O v/v) spectrum. Fig. S4. KASH2‐Ac isomer 1 1H–1H NOESY NMR (600 MHz, 90% H2O/10% D2O v/v) spectrum. Fig. S5. KASH2‐Ac isomer 1 1H–1H COSY NMR (600 MHz, 90% H2O/10% D2O v/v) spectrum. Fig. S6. KASH2‐Ac isomer 1 1H–15N HSQC NMR (600 MHz, 90% H2O/10% D2O v/v) spectrum. Fig. S7. KASH2‐Ac isomer 1 1H–13C HSQC NMR (600 MHz, 90% H2O/10% D2O v/v) spectrum. Fig. S8. KASH2‐Am isomer 1 1H–1H TOCSY NMR (600 MHz, 90% H2O/10% D2O v/v) spectrum. Fig. S9. KASH2‐Am isomer 1 1H–1H NOESY NMR (600 MHz, 90% H2O/10% D2O v/v) spectrum. Fig. S10. KASH2‐Am isomer 1 1H–1H COSY NMR (600 MHz, 90% H2O/10% D2O v/v) spectrum. Fig. S11. KASH2‐Am isomer 1 1H–15N HSQC NMR (600 MHz, 90% H2O/10% D2O v/v) spectrum. Fig. S12. KASH2‐Am isomer 1 1H–13C HSQC NMR (600 MHz, 90% H2O/10% D2O v/v) spectrum. Fig. S13. 1H–1H NOESY NMR spectra (600 MHz, 90% H2O/10% D2O v/v) of KASH2 peptides showing the NOEs consistent with the trans conformation. Fig. S14. Characterisation by RP‐HPLC and RP‐HPLC/MS of KASH2‐Ac produced with selective protection of the cysteine residues. Fig. S15. αH Secondary chemical shifts for KASH2 peptides. Fig. S16. A Bayesian phylogeny of DDH motifs. Fig. S17. Maximum likelihood phylogeny of the DDH motif. Table S1. Analysis of the backbone conformation of proline residues in KASH2 isomers. Table S2. Hydrogen bond restraints for KASH2. Table S3. Structural statistics for KASH2. [file FEB4-15-415-s001.docx]

**Table S1. Analysis of the backbone conformation of proline residues in KASH2 isomers.**

| **Peptide and Residue** | **β ^13^C chemical shift** | **γ ^13^C chemical shift** |
| --- | --- | --- |
| **Acid** |  |  |
| Isomer 1 - Pro 5 | 32.70 | 27.22 |
| Isomer 1 - Pro 10 | 32.72 | 28.01 |
| **Amide** |  |  |
| Isomer 1 - Pro 5 | 32.56 | 27.23 |
| Isomer 1 - Pro 10 | 32.70 | 27.97 |

**Table S2. Hydrogen bond restraints for KASH2*.**

| 10 PRO O 13 GLN H |
| --- |
| 14 TYR H 25 PHE O |
| 14 TYR O 25 PHE H |
| 16 GLN H 23 TYR O |
| 16 GLN O 23 TYR H |
| 18 LYS H 21 THR O |
| 18 LYS O 21 THR H |

*The same hydrogen bond restraints were used for both the acid and amide forms.

**Table S3. Structural statistics for KASH2.**

|  | **KASH2-Ac** | **KASH2-Am** |
| --- | --- | --- |
| **Experimental restraints** |  |  |
| Interproton distance restraints |  |  |
| *All* | 267 | 410 |
| *Intraresidue, \|i-j\|=0* | 79 | 123 |
| *Sequential, \|i-j\|=1* | 99 | 145 |
| *Medium range, 1 <\|i-j\| < 5* | 36 | 64 |
| *Long range, \|i-j\| >=5* | 53 | 78 |
| Disulfide-bond restraints (3 restraints per bond) | 6 | 6 |
| Dihedral-angle restraints | 41 | 41 |
| Hydrogen bond restraints (2 restraints per bond) | 14 | 14 |
| **R.m.s. deviations from mean coordinate structure (Å)** |  |  |
| Backbone atoms | 1.32±0.52 | 0.62±0.21 |
| All heavy atoms | 2.23±0.75 | 1.11±0.26 |
| Backbone atoms (13-26) | 0.35±0.25 | 0.20±0.10 |
| All heavy atoms (13-26) | 1.13±0.26 | 0.89±0.27 |
| **Ramachandran Statistics** |  |  |
| % In most favoured region | 80.5 | 94.4 |
| % Residues in additionally allowed regions | 19.5 | 5.6 |

**
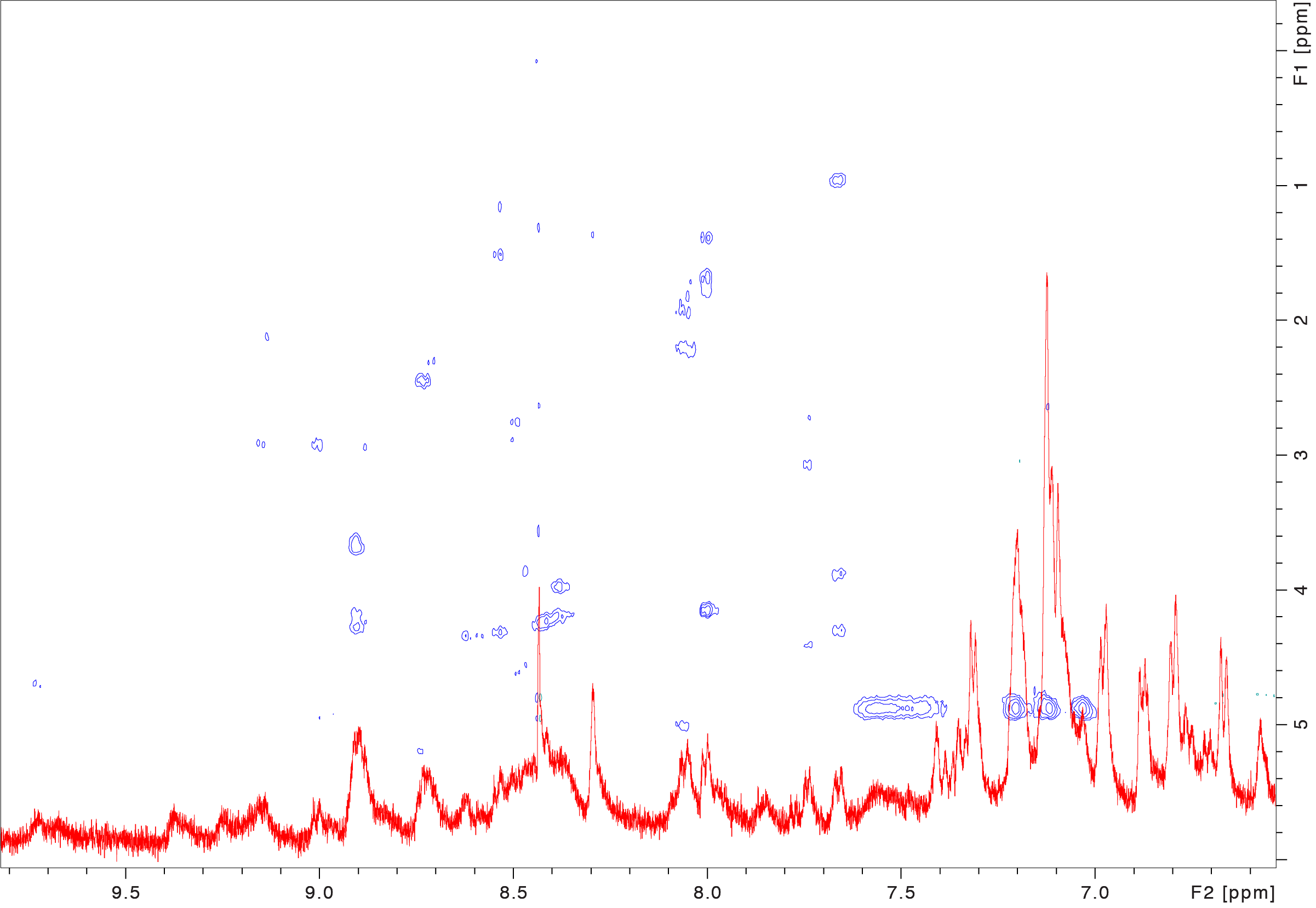
**

**Figure S1. KASH2-Ac at pH 5.5 ^1^H NMR (600 MHz, 90% H_2_O/10% D_2_O v/v) spectra.**

**
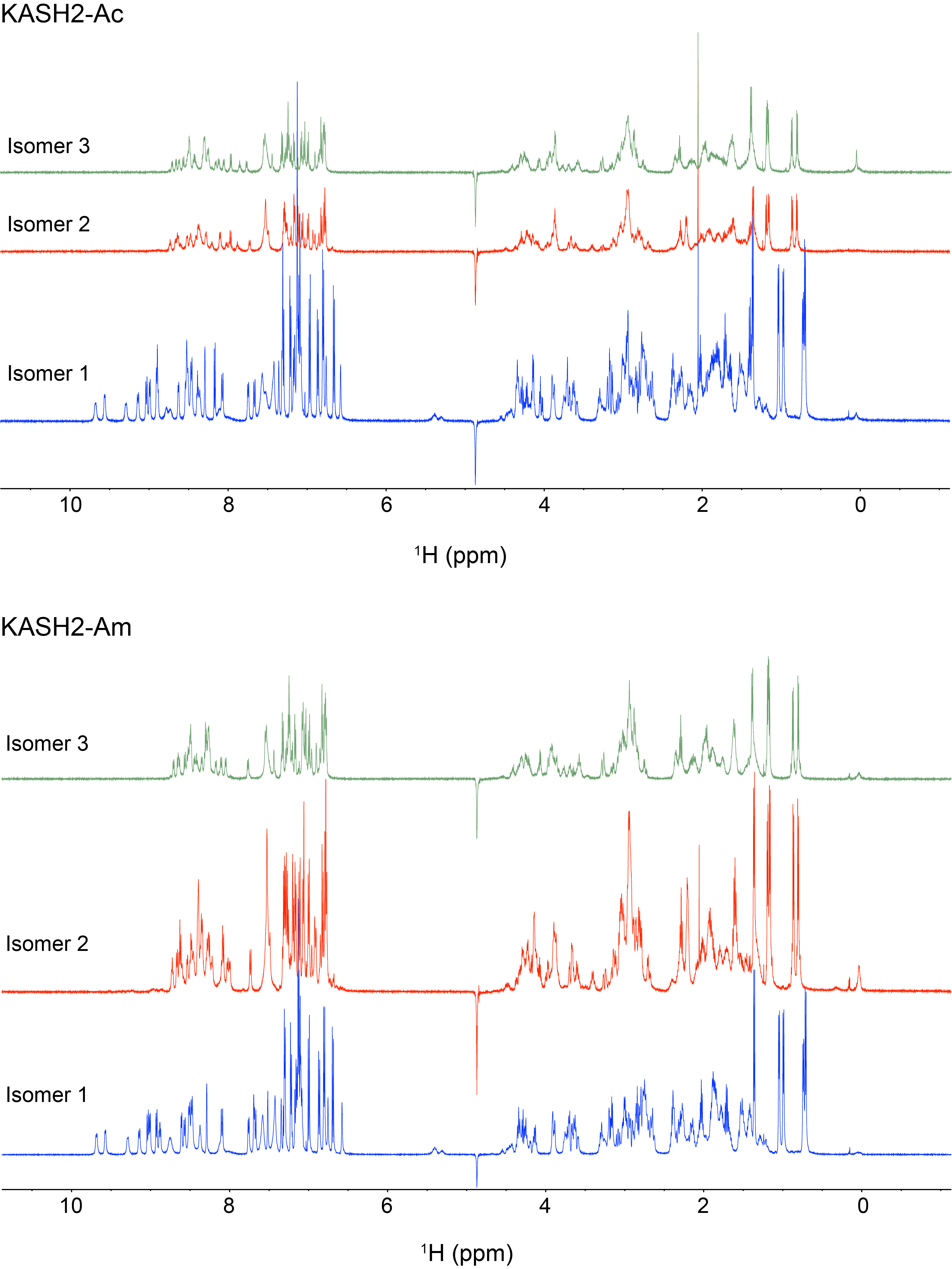
**

**Figure S2. KASH2-Ac, -Am ^1^H NMR (600 MHz, 90% H_2_O/10% D_2_O v/v) spectrum.**

**
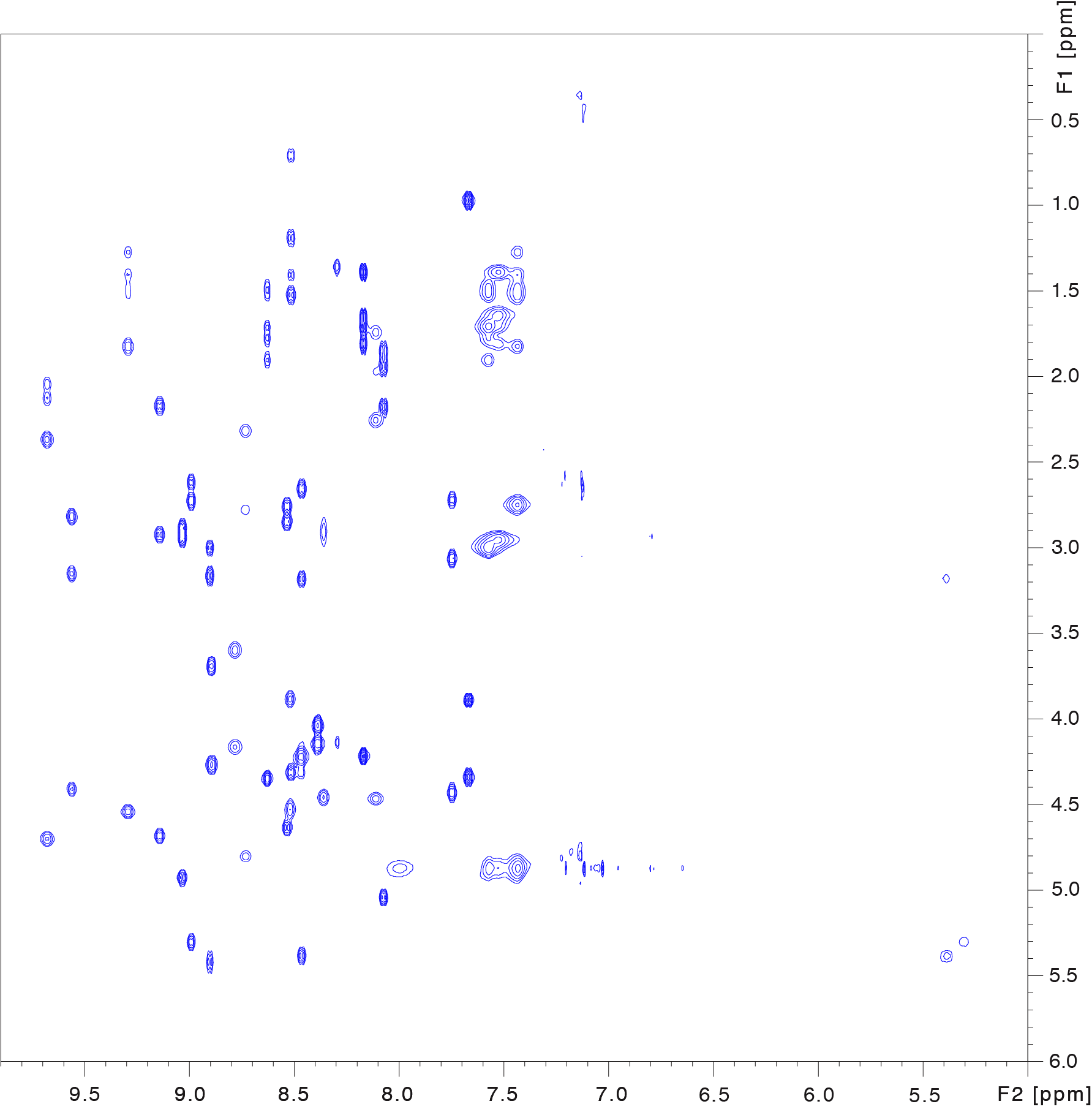
**

**Figure S3. KASH2-Ac isomer 1 ^1^H−^1^H TOCSY NMR (600 MHz, 90% H_2_O/10% D_2_O v/v) spectrum.**

**
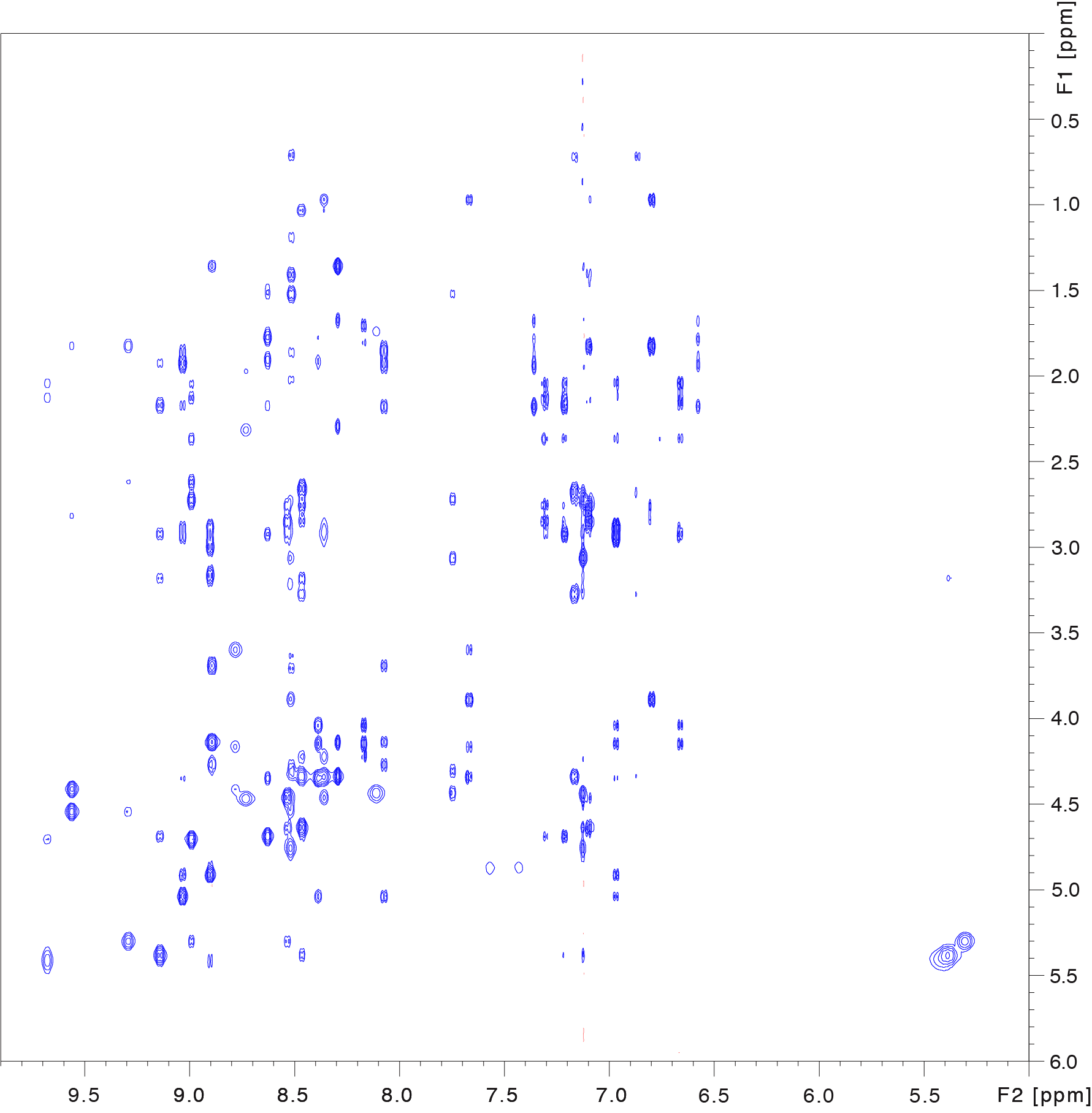
**

**Figure S4. KASH2-Ac isomer 1 ^1^H−^1^H NOESY NMR (600 MHz, 90% H_2_O/10% D_2_O v/v) spectrum.**

**
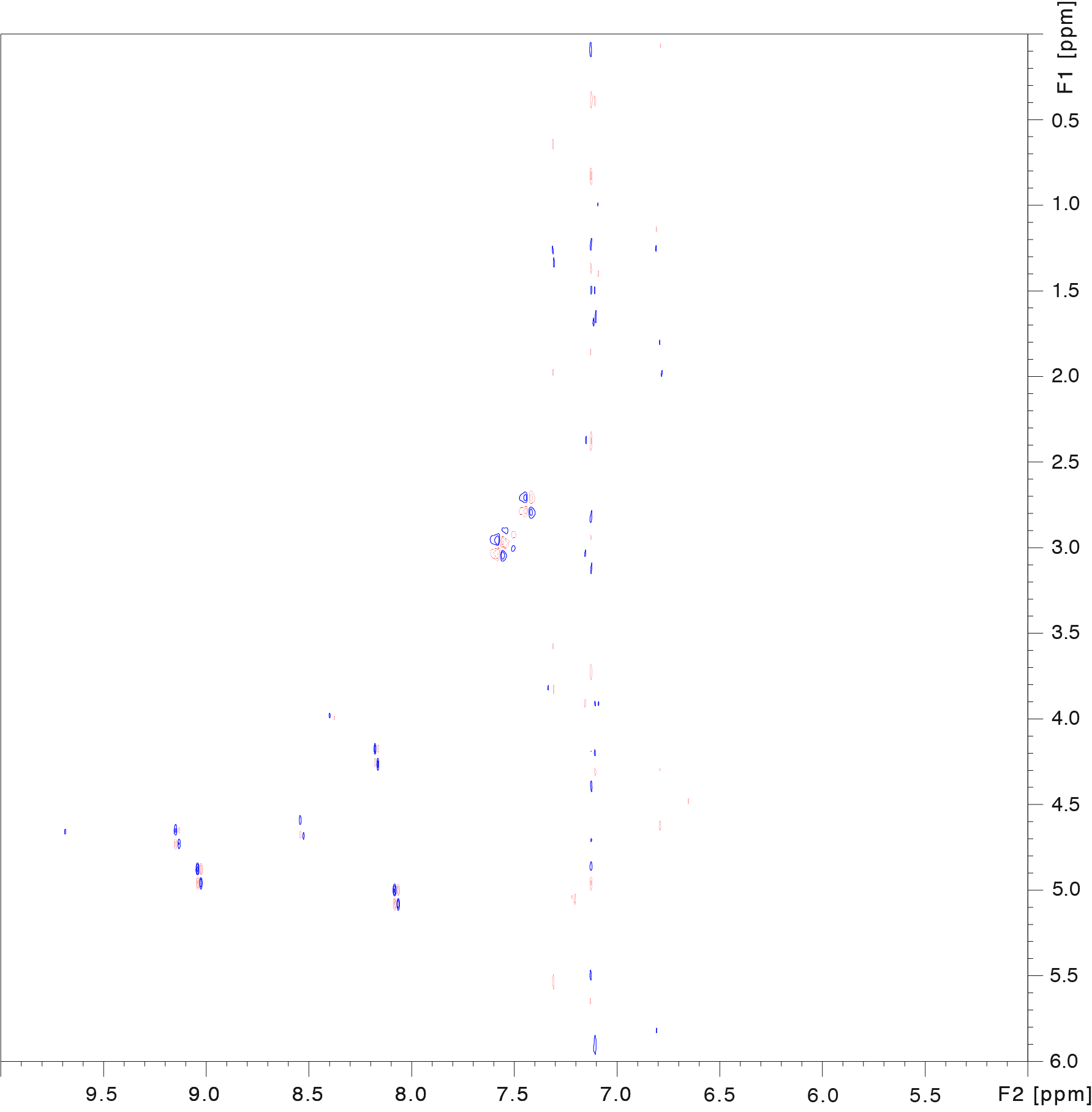
**

**Figure S5. KASH2-Ac isomer 1 ^1^H−^1^H COSY NMR (600 MHz, 90% H_2_O/10% D_2_O v/v) spectrum.**

**
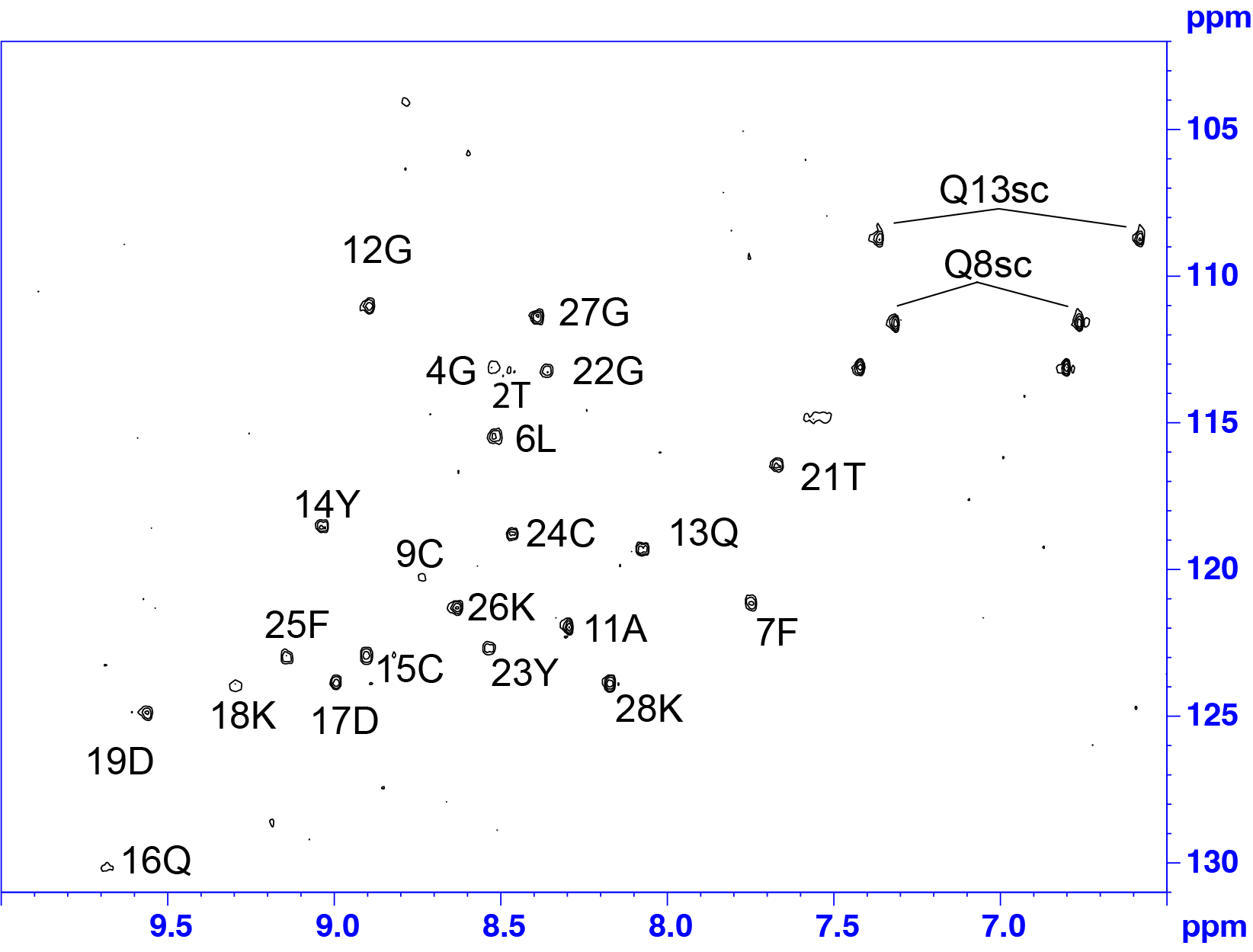
**

**Figure S6. KASH2-Ac isomer 1 ^1^H−^15^N HSQC NMR (600 MHz, 90% H_2_O/10% D_2_O v/v) spectrum.**

**
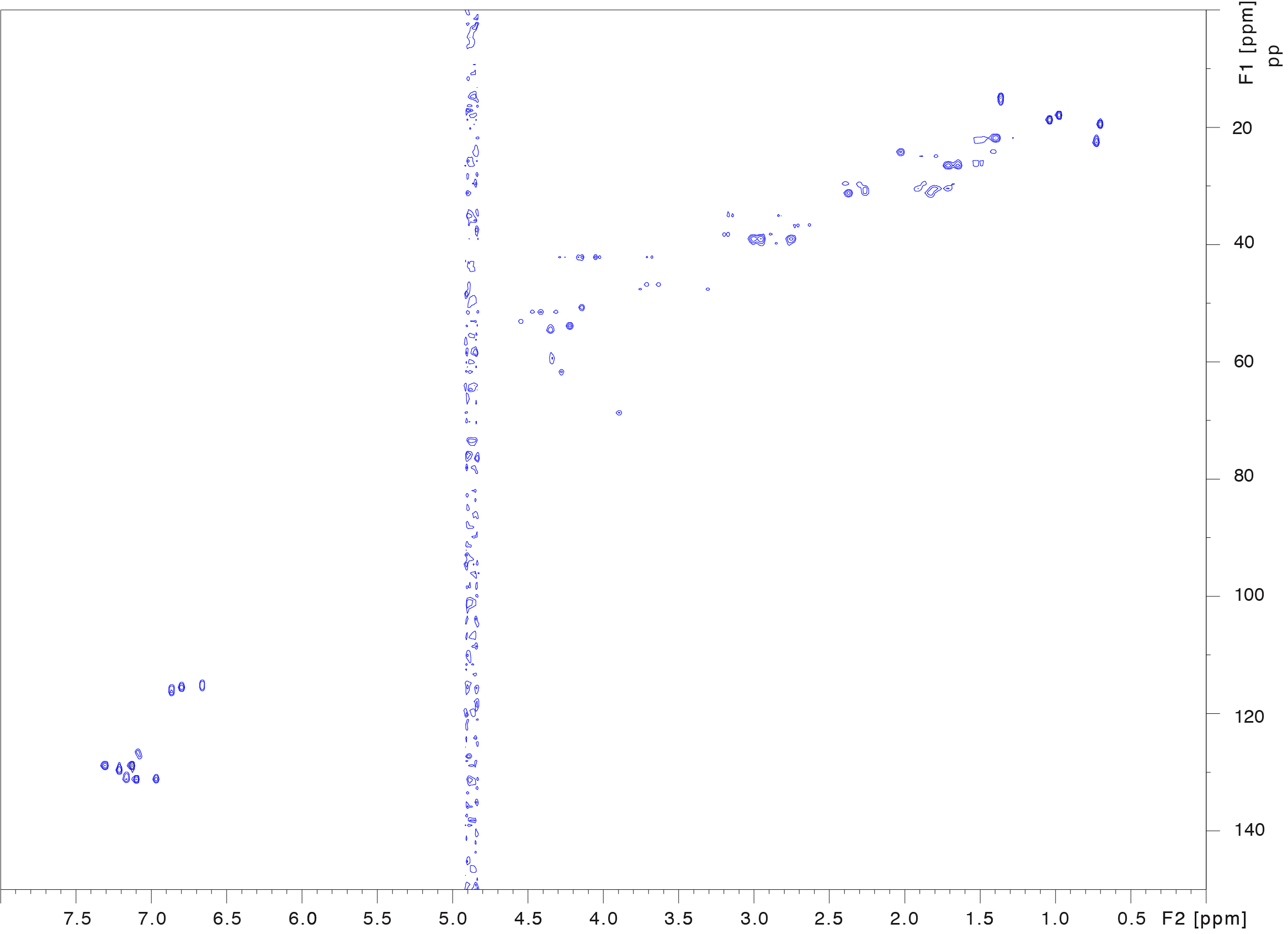
**

**Figure S7. KASH2-Ac isomer 1 ^1^H−^13^C HSQC NMR (600 MHz, 90% H_2_O/10% D_2_O v/v) spectrum.**

**
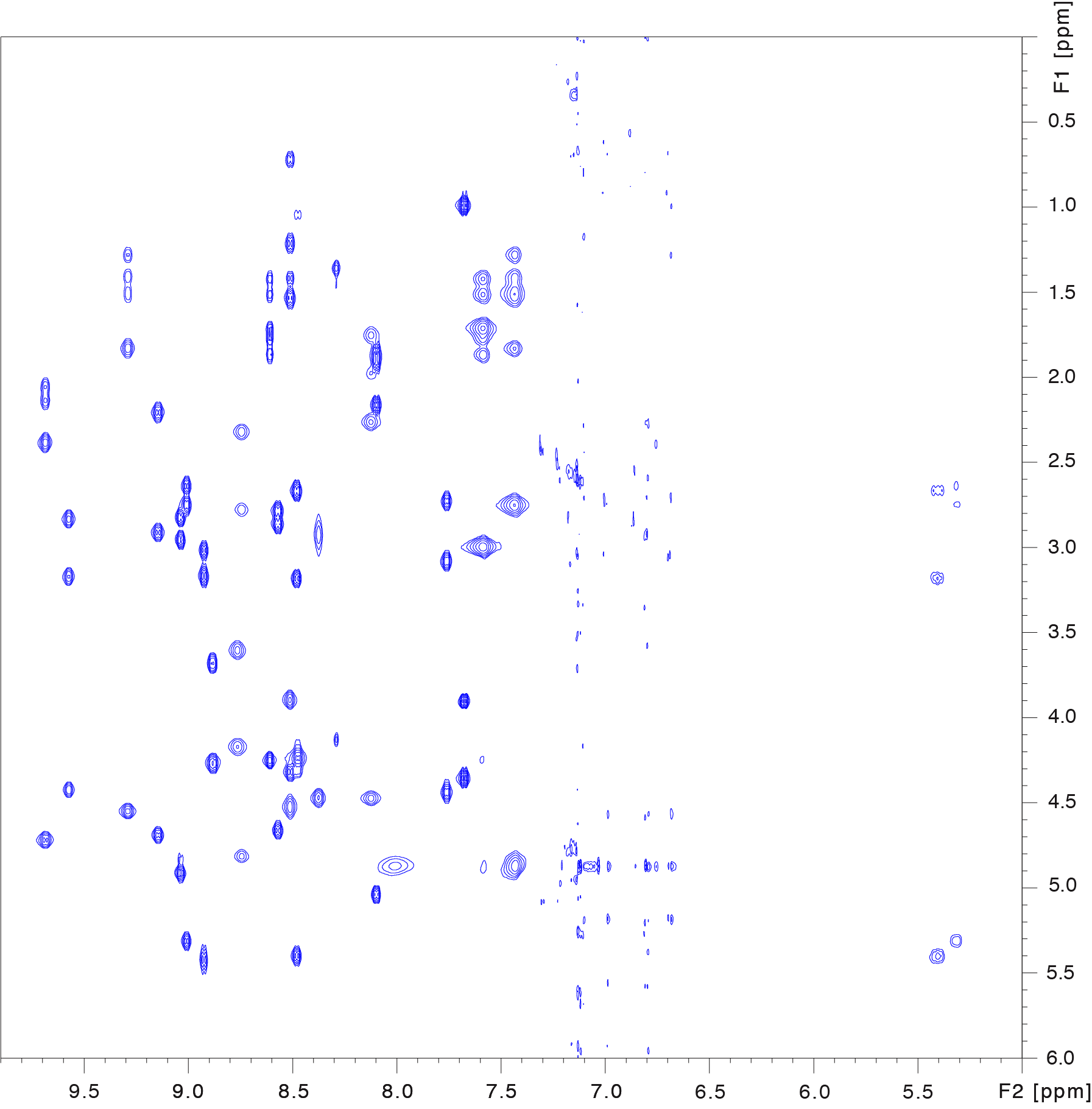
**

**Figure S8. KASH2-Am isomer 1 ^1^H−^1^H TOCSY NMR (600 MHz, 90% H_2_O/10% D_2_O v/v) spectrum.**

**
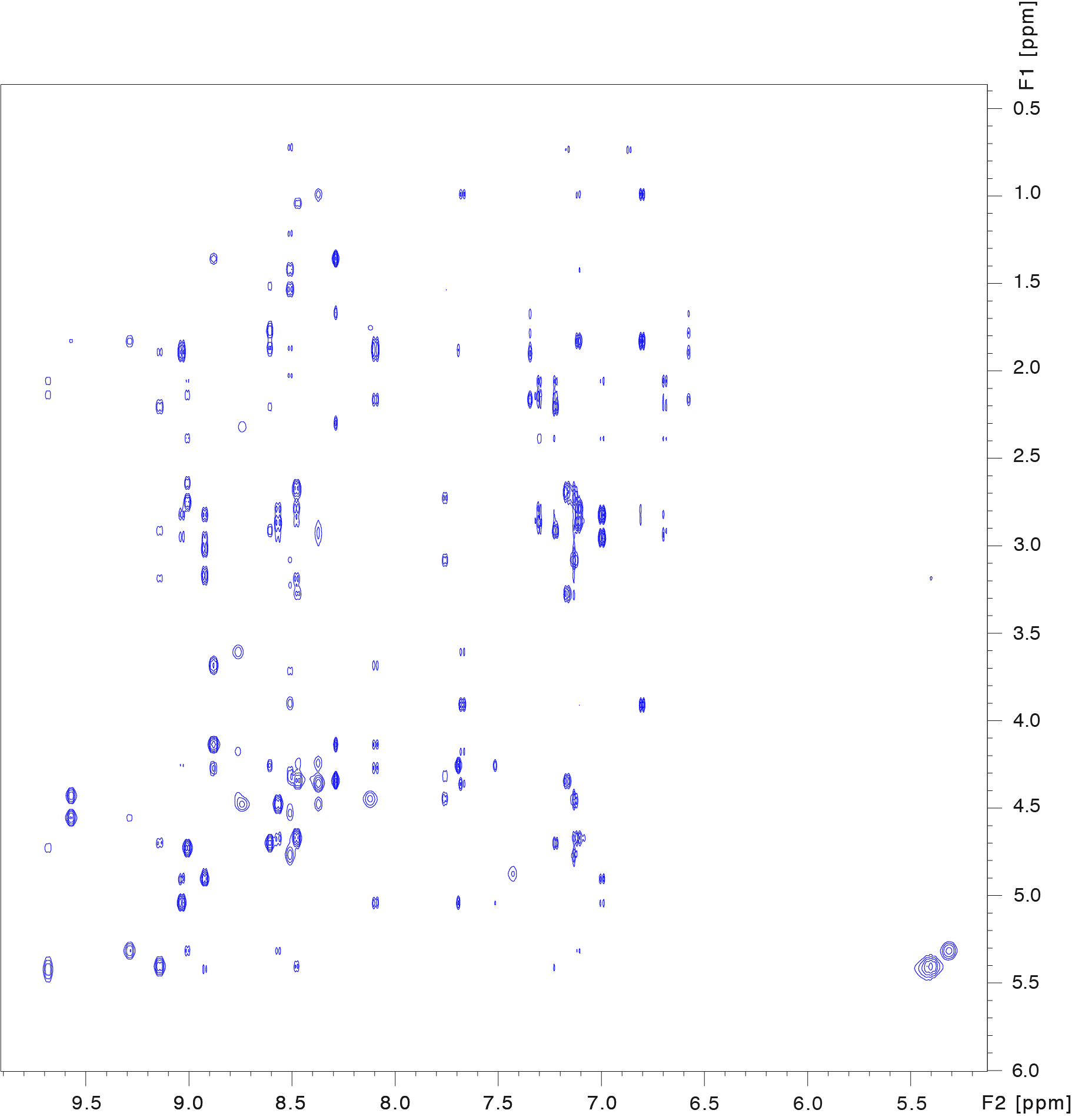
**

**Figure S9. KASH2-Am isomer 1 ^1^H−^1^H NOESY NMR (600 MHz, 90% H_2_O/10% D_2_O v/v) spectrum.**

**
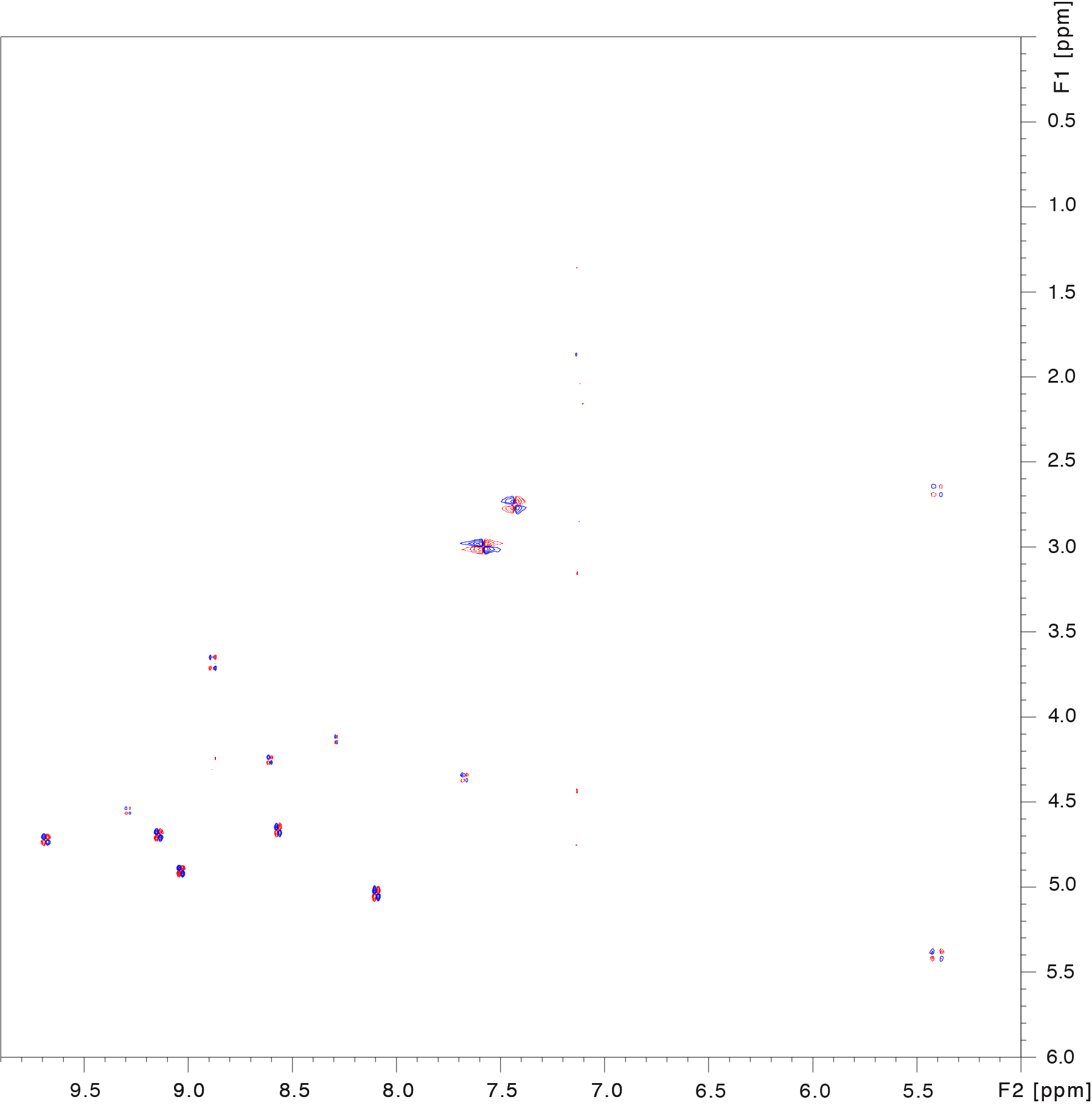
**

**Figure S10. KASH2-Am isomer 1 ^1^H−^1^H COSY NMR (600 MHz, 90% H_2_O/10% D_2_O v/v) spectrum.**

**
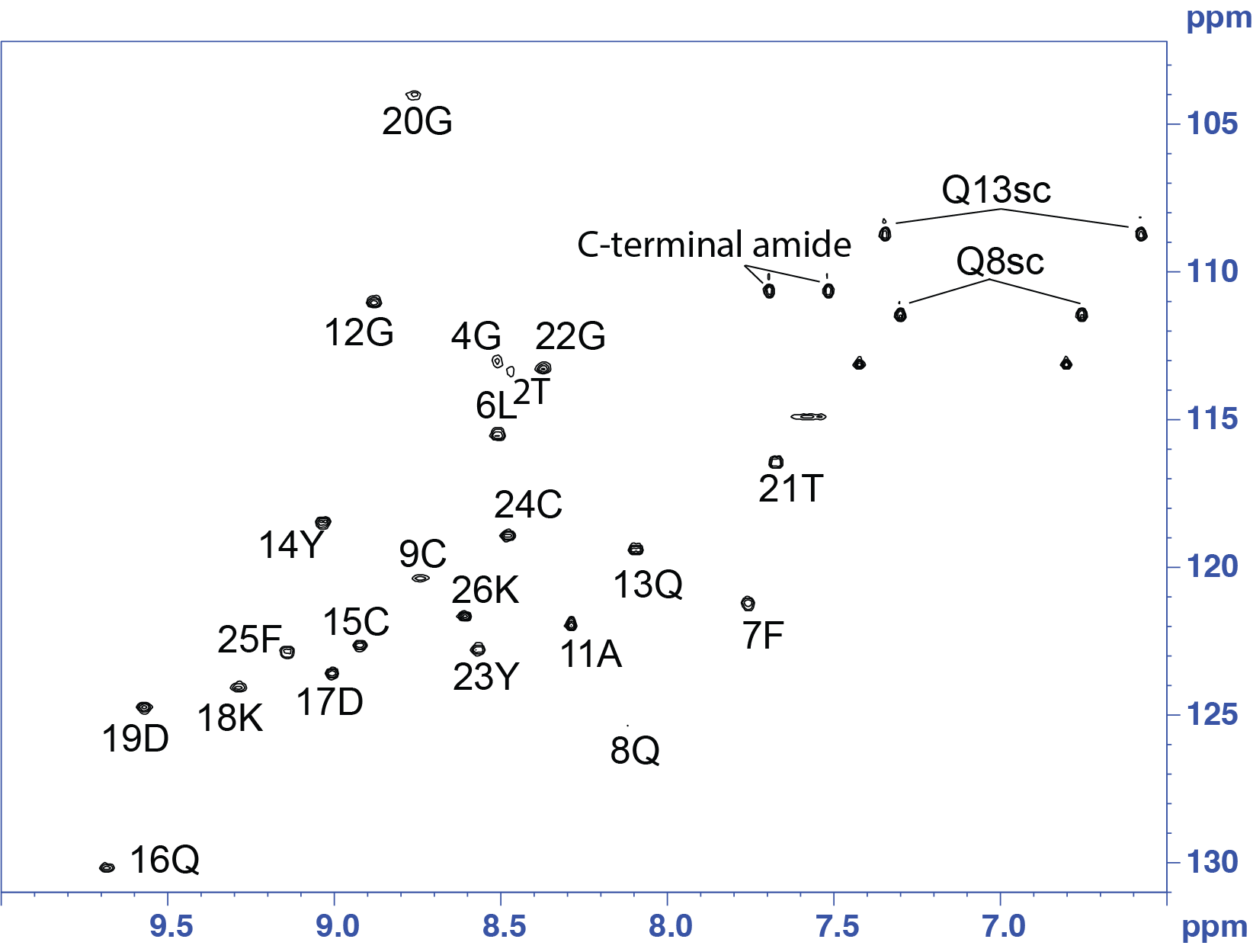
**

**Figure S11. KASH2-Am isomer 1 ^1^H−^15^N HSQC NMR (600 MHz, 90% H_2_O/10% D_2_O v/v) spectrum.**

**
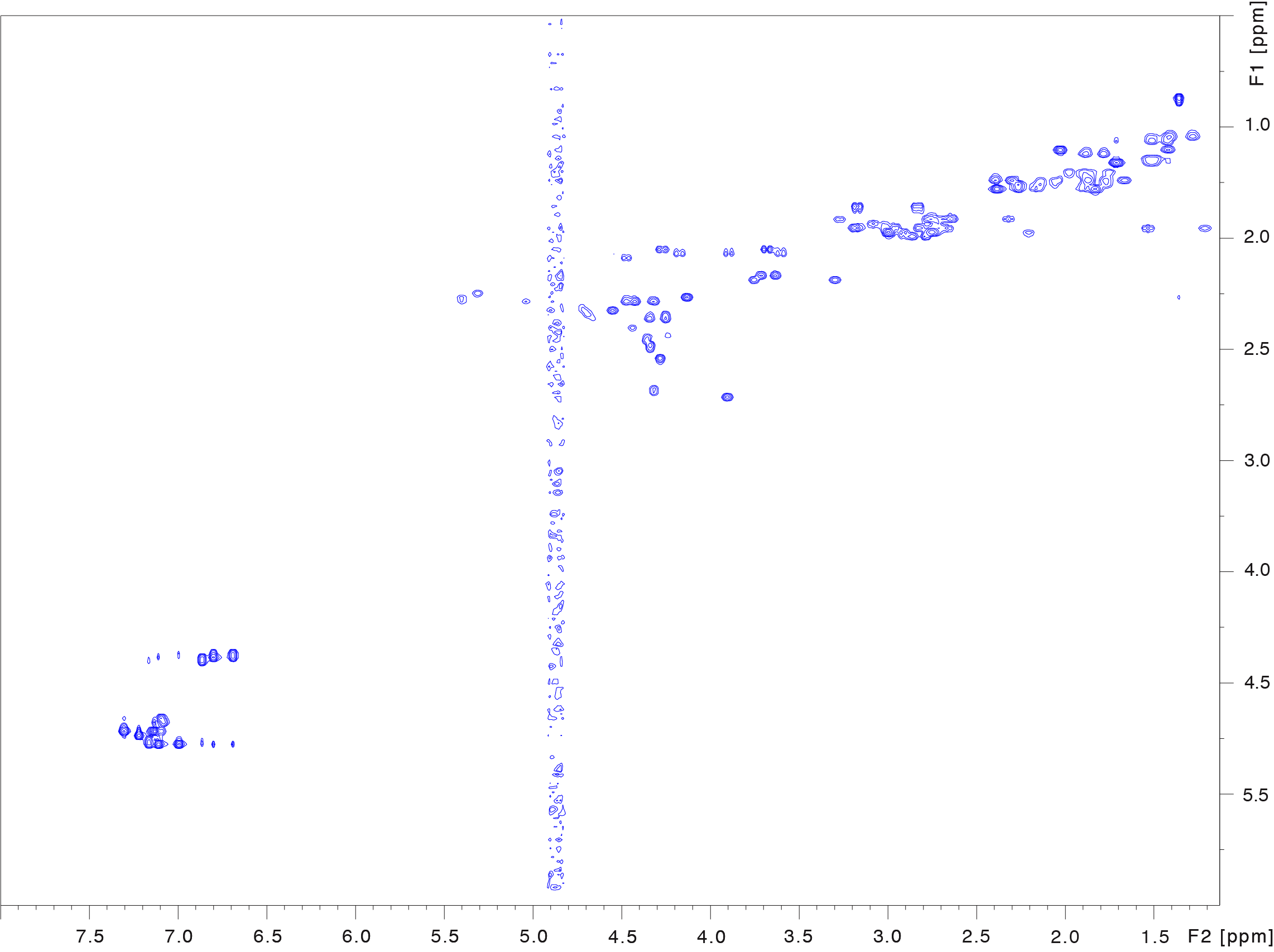
**

**Figure S12. KASH2-Am isomer 1 ^1^H−^13^C HSQC NMR (600 MHz, 90% H_2_O/10% D_2_O v/v) spectrum.**

**
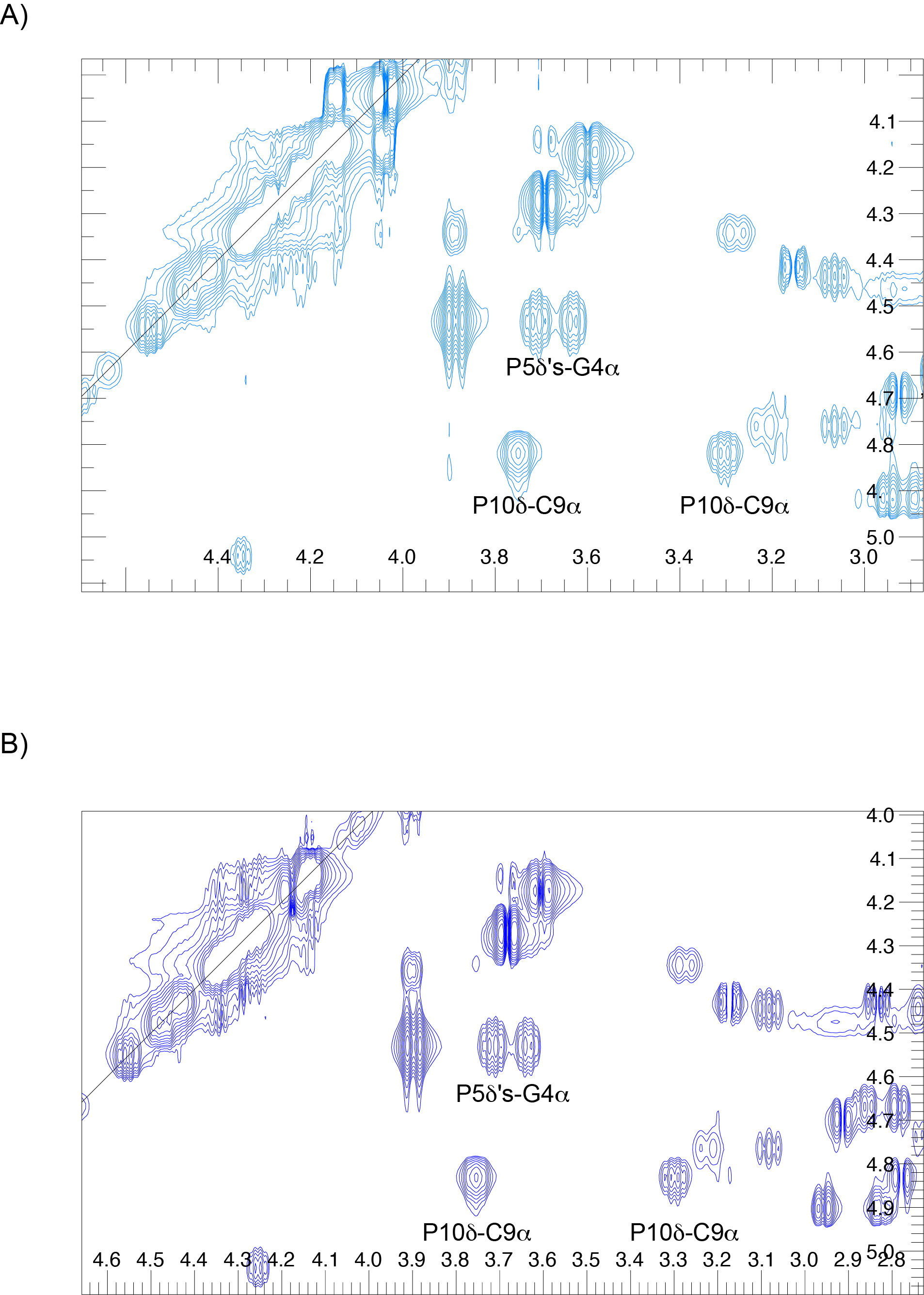
**

**Figure S13. ^1^H−^1^H NOESY NMR spectra (600 MHz, 90% H_2_O/10% D_2_O v/v) of KASH2 peptides showing the NOEs consistent with the trans conformation**

A) KASH2-Ac isomer 1 α-δ NOEs for Pro5 and Pro10 consistent with the trans-Pro conformation. B) KASH2-Am isomer 1 α-δ NOEs for Pro5 and Pro10 consistent with the trans-Pro conformation.

**Figure S14. Characterisation by RP-HPLC and RP-HPLC/MS of KASH2-Ac produced with selective protection of the cysteine residues.**

A) First oxidation (ACM groups still present). B) Final oxidation.

**
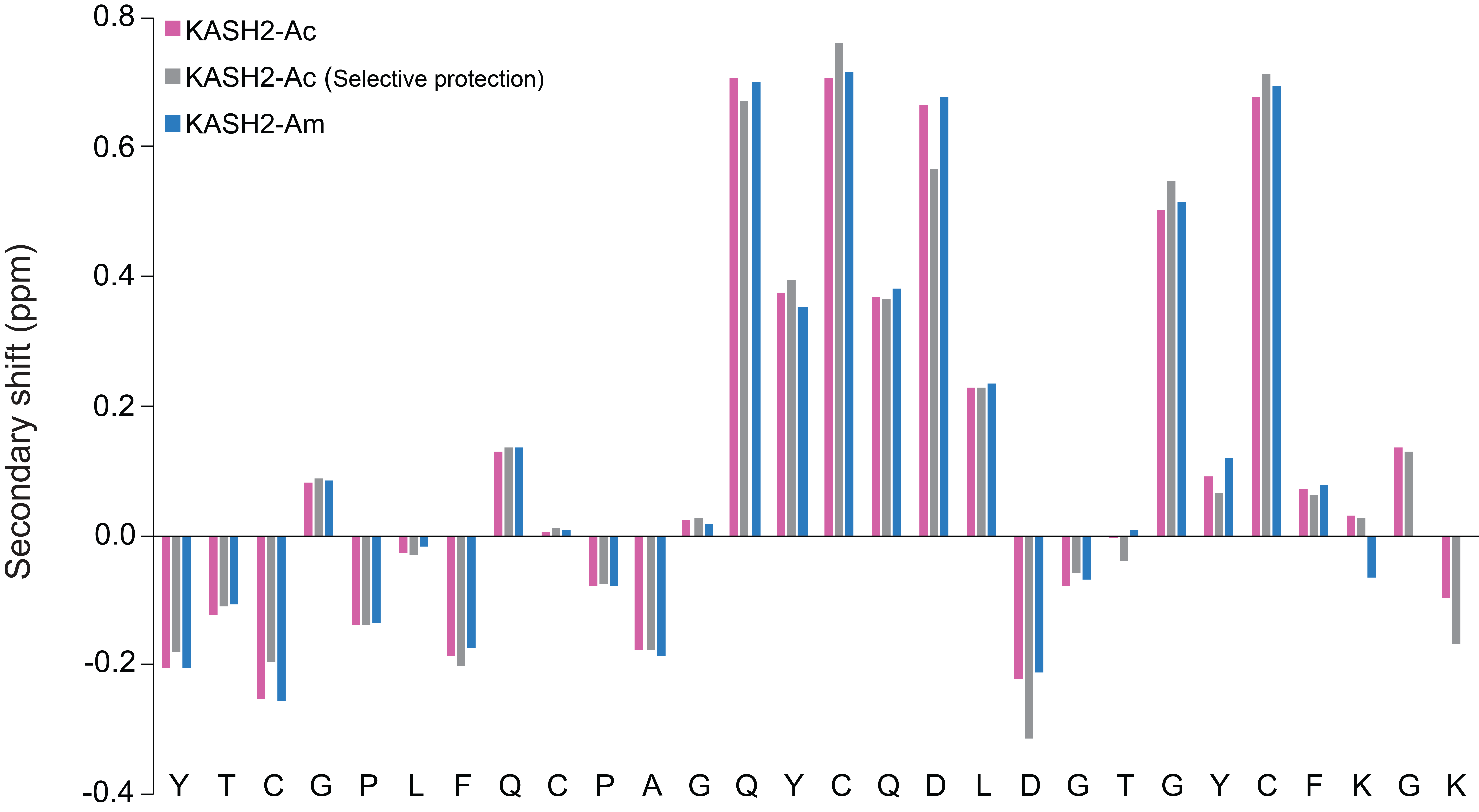
**

**Figure S15. αH Secondary chemical shifts for KASH2 peptides.**

The αH secondary chemical shift was calculated by subtracting the random coil ^1^H NMR chemical shifts previously reported by Wishart *et al*. from the experimental αH chemical shifts. The sequence of the peptides is given at the bottom of the diagram.





**Figure S16. A Bayesian phylogeny of DDH motifs.**

This figure highlights the Bayesian phylogeny of DDH scaffold. Here, the node supports are shown as Bayesian Posterior Probabilities (BPP), where nodes with BPP < 0.9 and those with BPP ≥ 0.90 are shown in light grey and thick black lines, respectively. DDH containing KASH2 from *A. rubens* is shown in red.

**

**

**Figure S17. Maximum likelihood phylogeny of the DDH motif.**

This figure represents the phylogeny of DDH scaffolds determined under a maximum likelihood inference. Here, node supports are evaluated with bootstrapping replicates (B), where nodes with B < 90 and those with B ≥ 90 are shown in light grey and thick black lines, respectively. DDH containing KASH2 from *A. rubens* is shown in red.
